# Improving the Identification of Phenotypic Abnormalities and Sexual Dimorphism in Mice When Studying Rare Event Categorical Characteristics

**DOI:** 10.1534/genetics.116.195388

**Published:** 2016-12-05

**Authors:** Natasha A. Karp, Ruth Heller, Shay Yaacoby, Jacqueline K. White, Yoav Benjamini

**Affiliations:** * Mouse Informatics Group, Wellcome Trust Sanger Institute, Cambridge, CB10 1SA, United Kingdom; † Department of Statistics and Operations Research, School of Mathematical Sciences, Tel Aviv University, Israel; ‡ Mouse Genetics Project, Wellcome Trust Sanger Institute, Cambridge, CB10 1SA, United Kingdom; § The Sagol School of Neuroscience, Tel Aviv University, 69978 Israel

**Keywords:** gene–phenotype map, mouse models, multiple testing, rare events, sexual dimorphism

## Abstract

Biological research frequently involves the study of phenotyping data. Many of these studies focus on rare event categorical data, and functional genomics studies typically study the presence or absence of an abnormal phenotype. With the growing interest in the role of sex, there is a need to assess the phenotype for sexual dimorphism. The identification of abnormal phenotypes for downstream research is challenged by the small sample size, the rare event nature, and the multiple testing problem, as many variables are monitored simultaneously. Here, we develop a statistical pipeline to assess statistical and biological significance while managing the multiple testing problem. We propose a two-step pipeline to initially assess for a treatment effect, in our case example genotype, and then test for an interaction with sex. We compare multiple statistical methods and use simulations to investigate the control of the type-one error rate and power. To maximize the power while addressing the multiple testing issue, we implement filters to remove data sets where the hypotheses to be tested cannot achieve significance. A motivating case study utilizing a large scale high-throughput mouse phenotyping data set from the Wellcome Trust Sanger Institute Mouse Genetics Project, where the treatment is a gene ablation, demonstrates the benefits of the new pipeline on the downstream biological calls.

COMPARING phenotyping results across genotypes, conditions, or treatments is a very basic research tool used widely in the life sciences. This paper was motivated by the statistical challenges raised in the analysis of data collected by the International Mouse Phenotyping Consortium (IMPC) (Brown and Moore 2012), which aims to phenotype knockouts for all mouse protein coding genes, as a premier model for understanding mammalian gene function. The freely available data (see www.mousephenotype.org) collected on each knockout line is extensive (Koscielny *et al.* 2013). For example, the current Wellcome Trust Sanger Institute (WTSI) phenotyping pipeline monitored 258 characteristics, 182 of them categorical. At the same time, the number of mice per knockout line, while at least seven females and seven males, is not much larger than 14. The analysis of the many categorical phenotypes on the relatively small number of mice per knockout group is challenging, as we elaborate below, so there is a need to improve the statistical methods used (Karp *et al.* 2012). In this work, we design an analysis pipeline specifically tailored to address the challenges posed by the IMPC data set, but the methodology developed is relevant to others as many studies have similar challenges.

The first challenge relates to the need to statistically assess for a treatment by sex effect. Historically, the majority of *in vivo* experiments studied one sex, typically focusing on males; to avoid complications from female estrogen cycles and pregnancy (Yang *et al.* 2006). However, it has been found that the majority of common diseases exhibit sex differences in prevalence, course and severity (Ober *et al.* 2008). Consequently, there has been growing concern over the imbalance in biomedical research (Kim *et al.* 2010; Flanagan 2014; Woodruff 2014). From inception, the IMPC pipeline focused on two sexes; however, to date, it has not tested explicitly for a genotype by sex interaction.

The second challenge arises from the rare event nature of many of the categorical phenotyping variables. For example, with traits such as head morphology (as expected/not as expected) or head bobbing (present/absent), the abnormal phenotypes are rarely seen in wild-type mice. Typically, the impact of a treatment on such data is assessed with Fisher’s exact test (FE) within IMPC, where both sexes are studied. The two sexes are processed separately with no statistical assessment of whether the genotype effect interacts with sex (White *et al.* 2013).

Another major challenge is the large scale of the problem. As phenotyping studies look at many characteristics simultaneously, this leads to concerns over how to manage the multiple testing problem where the simultaneous inferences can lead to the accumulation of false positives. Methods to manage the number of false positives arising from multiple testing can be categorized into those that control the probability of at least one false positive (family-wise error rate) or those that control the expected proportion of false positives out of all rejected hypotheses (false discovery rate, FDR). The current strategy utilized by IMPC is to use an *ad hoc* stringent significance threshold (*P* < 0.0001). For rigorous hypothesis-generating studies such as high-throughput phenotyping, attention focuses on the control of the FDR to maximize sensitivity but manage false positives (Benjamini and Hochberg 1995; Reiner *et al.* 2003). Here too, the control of FDR across all hypotheses within a knockout line and all lines together, or within each line separately, is appropriate. However, when the control is within each line separately there is a need to adjust the threshold used within families to ensure overall control over false positives, *e.g.*, control of the average FDR across all families (Benjamini and Bogomolov 2014).

The final challenge arises from the discreteness of the test statistics resulting from the data being categorical. For discrete test statistics, it is possible that *P*-values can take, for example, the values 0.062 or 0.043 but not the 0.05 in between. Since the *P*-value has to be ≤ 0.043 for a result to be significant at 0.05, the actual error rate can be can be much smaller than the desired level, further limiting power. Furthermore, the actual error rate for a multiple testing method that does not take into account the discreteness can be even lower. Indeed, multiple testing with discrete data is an active area of research (Kulinskaya and Lewin 2009; Ahmed *et al.* 2010; Heyse 2011). One strategy to manage multiple testing is to reduce the family size by using a filter independent of the test statistic outcome (Bourgon *et al.* 2010). For categorical data, the use of potential filters to remove hypotheses that are unable to reach statistical significance has been proposed (Tarone 1990; Gilbert 2005).

In this manuscript, we consider various methods for testing both a treatment effect and an interaction between the treatment and sex for categorical data. The goal was to identify a pipeline that maximizes sensitivity while considering the multiplicity and the discreteness issues. In particular, we first assess the impact of genotype ablation, which can arise either from change in both sexes or one sex, and then assess whether the effect of genotype depends on sex, but only if the genotype effect was found to be statistically significant.

We chose this two-step process to reduce the multiple testing problem at stage 2 to maximize sensitivity, essential when one considers that statistical testing for interactions is a low-power activity (Greenland 1983). After simulations to compare various statistical techniques, we propose a methodology and demonstrate its use on a large multi-gene, multi-screen data set.

## Materials and Methods

### Generation of data

Supplemental Material, File S1 provides detailed explanation of how the data were generated. In summary, the analysis uses data generated by a high-throughput phenotyping pipeline, where a mouse is characterized by a series of standardized and validated tests that were chosen to cover a variety of disease-related and biological systems. Phenotyping data are collected at regular intervals on age-matched wild-type (control) mice of equivalent genetic backgrounds. Cohorts of at least seven mice of each sex per knockout line were generated.

### Assessing the role of batch variation

Firth’s bias reduction logistic regression (R package: logistf) was used to test the role of batch (assay date) using a likelihood ratio test between a test model (*Y ∼* sex *+* batch) and null model (*Y ∼* Sex) for each trait of interest (*n* = 182) in wild-type data from the MouseGP Pipeline. The measures of significance were adjusted for multiple testing using the Benjamini–Hochberg procedure (BH, Benjamini and Hochberg 1995) to control the FDR at 5%.

### Statistical assessment of stage 1: genotype effect

Four statistical methods were evaluated for stage 1 testing of the genotype effect (see File S2 and Table 1 for additional mathematical details). Full implementation can be seen within the associated scripts. (i) FE is used to compare the abnormality rates in wild-type and knockout for each sex separately. The lowest *P*-value from the two tests multiplied by 2 is the *P*-value for this test. Note that FE, as implemented within PhenStat, is the currently used method in IMPC (Kurbatova *et al.* 2015). (ii) The mid method for discrete test statistics, that takes the standard *P*-value for the test minus half the difference between it and its nearest lower possible value (Lancaster 1961; Hwang and Yang 2001), is used to modify the FE test designed to increase power in the face of its discrete distribution (Rothman *et al.* 1998) (see File S2 for the formula). (iii) Firth’s bias reduction logistic regression (Heinze and Schemper 2002) employs a likelihood ratio test comparing the null model (*Y ∼* sex) with the test model (*Y ∼* genotype *+* sex *+* genotype*sex), but is modified to address rare events (the LR_G method). (iv) The one-sided Cochran–Mantel–Haenszel test compares the proportion of rare events difference between the knockout and wild-type groups, stratified by sex (Hollander *et al.* 2014) (see File S2 for the formula). An adaptation to increase the sensitivity uses the mid-*P*-value (the MH_ mid method).

**Table 1:** The statistical methods considered for testing genotype and genotype*sex effect

Testing	Methods	Summary
Genotype	FE	For each sex, a FE test compares the abnormality rate between wild-type and knockout mice; the lowest *P*-value multiplied by 2 is returned, due to multiple testing arising from havinga test for each sex
	FE_mid	For each sex, the mid-*P*-value of a Fisher’s exact test which compares the proportions between wild-type and knockout is calculated; the lowest *P*-value multiplied by 2 is returned, due to multiple testing arising from having a test for each sex
	MH_mid	The Mantel–Haenszel mid-*P*-value tests for an association between genotype and the abnormality rate across the two sexes
	LR_G	A likelihood ratio test is used to compare a null model with a genotype model built using biased reduction logistic regression
Genotype*sex	Zelen	The Zelen mid-*P*-value test for an interaction between genotype and sex by testing for homogeneity of association
	LR_I	A likelihood ratio test is used to compare a null model with a test model which includes an interaction term built using biased reduction logistic regression
	LR_KO	Using the knockout data only, a likelihood ratio test is used to compare a null model with a test model which includes sex built using biased reduction logistic regression
	FE_KO	Using the knockout data only, the mid-*P*-value of a FE test for comparing proportions between the sexes

Detailed statistical explanations are available in the *Materials and* *Methods*. FE, traditional Fisher’s exact test; FE_mid, Fisher’s exact test mid-*P*-value; MH_mid, Mantel–Haenszel mid-*P*-value; LR_G, biased reduction logistic regression; Zelen, Zelen mid-*P*-value; LR_I, biased reduction logistic regression with an interaction term; LR_KO, biased reduction logistic regression using knockout data only; FE_KO, Fisher’s exact test mid-*P*-value using knockout data only.

To assess the size of the biological effect, first the differences in two binomial proportions were calculated for each sex and the 95% C.I. calculated utilizing Newcombe’s recommended method 10 (which is based on the Wilson score method for single proportions, without continuity correction) via the ci.pd function of the R Epi package (Newcombe 1998). Then, the average of the two differences was calculated and a conservative C.I. determined by selecting was computed, based on the two C.I., to have as its lower bound the minimum of the two lower bounds and as its upper bound the maximum of the two upper bounds.

### Statistical assessment of stage 2: interaction effect

Four statistical methods were evaluated for stage 2 (see Table 1 and File S2 for additional mathematical details). Full implementation can be seen within the associated scripts. The first two methods, noted as LR_I and Zelen, test the interaction between genotype and sex. The LR_I method again uses Firth’s bias reduction logistic regression of (iii) in stage 1, but this time to compare the null model (*Y ∼* sex *+* genotype) with a test model (*Y∼* genotype *+* sex *+* genotype*sex). The second method, developed by Zelen (Hollander *et al.* 2014), is a nonparametric two-sided test, assessing whether the odds ratio in males differs from the odds ratio in females (where the odds ratio is ratio of the odds of an abnormality in the knockout and the odds of an abnormality in the wild-type).

The last two methods, LR_KO and FE_KO, assess the impact of sex on the abnormality rate within knockout data only. This hypothesis can be viewed as assessing the “interaction” if the assumption that the background rates are the same across sexes is true. As the abnormality rate in the wild-type mice is very low, it may not be possible to reject the hypothesis of interaction, but the hypothesis of equal rates in male and female knockout mice may be rejected if there are big differences in the rates in the knockout mice. The third method assesses the impact of sex on abnormality rate within the knockout data, again using Firth’s bias reduction logistic regression (the LR_KO method) with a likelihood ratio test that compares a test model (*Y* ∼ sex) with a null model where *Y* is constant. The fourth method uses the FE test with the mid-*P* method modification, method (ii) of stage 1, but this time comparing the proportions across the two sexes seen within the knockout data only (the FE_KO method).

To estimate the biological effect, the differences in two binomial proportions was calculated and the 95% C.I. calculated utilizing Newcombe’s recommended method 10.

### Assessing reliability: control of type-one error rates in the absence of a genotype effect

Assessment under the global null was done by simulating control data to give 100 dependent variables with 2 years of data with seven males and seven females collected weekly, by random independent sampling from binominal distributions with defined abnormality rates. The 100 abnormality rates were selected by randomly sampling 100 variables from a WTSI skeletal screen (*n* = 46 variables), eye morphology (*n* = 26 variables), and Neurological and Morphology Phenotypic Assessment (NAMPA) (*n* = 114 variables) screens, and taking the observed abnormality rate seen in the wild-type B6N data from the Mouse GP pipeline, which ran from August 2009 until February 2012 and collected control data weekly for seven males and seven females. Resampling studies were conducted to assess the rate of type-one errors in the absence of any genotype effect (the global null hypothesis). Mice (seven males and seven females) were sampled from the simulated data at random without replacement and relabeled as knockout. The resulting data set was then examined for statistically significant differences between the wild-type and artificially generated knockout mice by the statistical pipeline under consideration. The number of iterations was 15,000.

The process described above was repeated using the “real” control data taken from the wild-type–knockout eye screen data set for the 26 traits studied. The eye screen was selected as a data set with the largest range of abnormality rates in the control data. The number of iterations was 1250.

### Assessing reliability: control of type-one error rate for stage 2 in presence of a genotype effect

Wild-type baseline data, equivalent to two years of weekly collection for seven male and seven female mice, for traits with a baseline abnormality rate of 0.05, were generated from a binomial distribution for each sex in each of 15,000 iterations. Seven males and seven females were randomly selected (in each iteration) to represent knockout mice and the same effect was added to both sexes. The artificially generated wild-type–knockout data sets were then statistically processed to assess the type-one error rates of the stage 2 test with various significance thresholds (0.05, 0.025, 0.01, and 0.001). The simulation was conducted for various genotype effect sizes (0, 0.1, 0.2, …, and 0.9). The assessment of the stage 2 type-one error rate was done with and without filtering for significance at stage 1 using various significance filters (0.001, 0.01, 0.025, and 0.05).

### Assessing the statistical power

The statistical power was assessed via simulations. Wild-type baseline data, equivalent to two years of weekly collection of seven males and seven females, for five traits with varying baseline abnormality rate (0, 0.01, 0.025, 0.05, and 0.075), were generated from a binomial distribution for each sex in each of 15,000 iterations. Seven males and seven females were randomly selected (in each iteration) to represent knockout mice and signal was added. The statistical power was assessed for the various statistical tests. The statistical power at stage 1 was assessed for a variety of main effects (0, 0.1, 0.2, and 0.3) in the presence of a variety of interaction effects (0, 0.2, and 0.35). The statistical power at stage 2 was assessed with and without filtering the significant hypotheses at stage 1 (at level α = 0.05). The implementation can be seen in the associated scripts.

### Managing the multiple testing problems

For discrete test statistics, as addressed here, the smallest attainable *P*-value can be above the level of testing. Therefore, filtering hypotheses for which statistical significance cannot be attained substantially reduces the multiple testing burden and has been proposed to increase sensitivity (Tarone 1990). The filtering is implemented by computing for each hypothesis the smallest possible *P*-value, termed “α star,” while keeping the marginal counts fixed, and then pursuing only tests that have α stars below a predefined threshold while preserving the sums of wild-type and knockout rare events for each sex.

Two FDR type methods were considered for the multiple testing problem within a set of hypotheses at stage 1 (genotype testing) or stage 2 (sexual dimorphism). The BH procedure was first used to control the FDR across all hypotheses being considered. To control the average FDR across gene families, the Benjamini and Bogomolov (BB) method was implemented (Benjamini and Bogomolov 2014). This is a two-step process; in the first step we screen the families for which a detailed analysis is desired, and then test within the selected families to control an average error rate. For the first step, selection of families, we compute the number of families with at least one discovery using a within-family BH procedure at level 0.05. Let *R* be the number of families selected. In the second step, we adjust for the selection by declaring findings as significant only if they were discovered using a within-family BH procedure at level R*0.05/m, where m is the number of families.

### Data availability

The manuscript has been prepared as a reproducible study and, as such, all data and scripts have been given a unique Digital Object Identifier via Zenodo (http://doi.org/10.5281/zenodo.164696). The raw data are available via the IMPC database (www.mousephenotype.org). 

## Results and Discussion

### Categorical phenotyping data characteristics

Exploration of the control data from the WTSI Mouse Genetics Project pipeline allowed a detailed assessment of characteristics of variables typically monitored in phenotyping studies. We found that the majority of the variables are rare event monitoring (Figure S1): 82% of the variables had an abnormality rate < 1% and the median abnormality rate was 0.04% in wild-type mice.

With continuous phenotyping data, batch-to-batch variation has been identified as a significant source of variation (Karp *et al.* 2012), where batch encompasses many sources of variation including litter, operator, cage, and reagent etc. For the majority of categorical variables (95.6%), there was no evidence of batch-to-batch variation (Table S1). This has an advantage, in that we can combine data from separate batches when collected with the same meta-data values (*e.g.*, genetic background, standard operating procedure, and diet), which will increase sensitivity as there will be a larger effective sample size to estimate the abnormality rate in the control data. Within the WTSI data, the median number of mice from a litter was two and, on average, the knockout data for a gene and zygosity arose from a mean of 5.7 litters. With the high number of litters it would be challenging to model litter. Further work in this manuscript will focus on methods that assume that batch-to-batch and litter variation is negligible.

### Considerations affecting the choice of statistical methods

We identified three possible techniques for comparison to the FE test, which is currently used in IMPC, for the stage 1 testing for a genotype effect (Table 1). The nonparametric Mantel–Haenszel (MH) test has the benefit that it aggregates the tests of the genotype effect across the two sexes. This test will be more sensitive, provided the direction of associations for the two sexes is always the same, which is a safe assumption when monitoring rare event traits in search for an increase in “abnormal.” The discrete nature of the FE and MH tests has led to the adaptation of the techniques to return a mid-*P*-value to improve the sensitivity. Rare event data will frequently lead to separation in the data where the genotype becomes a highly predictive risk factor and this, with the traditional logistic regression, will lead to poorly estimated model parameters (Heinze and Schemper 2002; Heinze 2006). The Firth’s bias reduced logistic regression (Heinze 2006) was designed as an adjustment to logistic regression that addresses this exact issue.

For stage two, we identified four approaches for investigation (Table 1). The parametric Firth’s biased reduction logistic regression methodology can be used to assess the impact of sex from two angles. First, utilizing all data, we can test the role of a genotype by sex interaction term (LR_I). Second, with just the knockout data, we can assess the role of sex (LR_KO). The advantage of the LR_KO test is that it can have good power if the abnormality rates differ across sexes in the knockout groups in a situation of very low background abnormalities rates (where there is a large probability of observed zero abnormalities in the wild-type). In contrast, the LR_I test has no power to detect an interaction in such a situation. A potential weakness of the LR_KO test arises as it only analyses the knockout data; therefore, it will not have the potential to detect an interaction when the abnormality rate in the knockouts is similar, but there is a difference in abnormality rate in the wild-type population. Other nonparametric tests can also be used, and can benefit from the mid-*P*-value modification to increase sensitivity. The FE can be used to compare the proportion rates between sexes of the knockout data (FE_KO), while the nonparametric Zelen test directly tests for a genotype*sex interaction (Zelen 1971).

The fact that the candidate lines for stage 2 turned out to be significant at stage 1 could harm the validity of the *P*-values in stage 2. However, this is not the case when the MH_mid is used at stage 1, and in stage 2 either the Zelen test of FE_KO, which utilizes the knockout data only, is used because they are conditional on the total number of rare events in stage 1. Therefore, we expected that, for the other tests at stage 2, the *P*-values would be approximately valid as well and checked this property using a simulation study.

Following hypothesis testing, the effect size (the magnitude of the genotype effect) needs estimating. Odds ratios are frequently used with categorical data; however, they are not suitable with rare event data as this can lead to the denominator being close to zero (or zero) giving very large estimates (even infinity) but with low precision. We propose examining the percentage change in abnormality rate with C.I. estimated using the Newcombe recommended method (Newcombe 1998). We selected this method as it was recommended for highly unbalanced designs (Prendergast and Staudte 2014). In the presence of sexual dimorphism, the percentage change in abnormality rate will be dependent on the sex. Therefore, we suggest estimating the average effect size across the sexes by estimating the effect size separately for each sex independently, and then calculating an average effect size. A conservative C.I. for this average can be estimated by taking the lowest and highest confidence bound from the independent estimates; however, in the presence of sexual dimorphism this can lead to wide estimates.

The following sections consider the power and type-one error rate control to enable selection of a robust analysis pipeline.

### Assessing reliability of the statistical calls: control of the type-one error rate

A simulation study assessed the behavior of the methods under the null hypothesis for both stage 1 and 2. Simulated wild-type data, representing 100 typical traits, were sampled to build knockout data sets that were compared back to the wild-type, and the process repeated to assess how many false calls would occur in a situation of no biological effect.

**Figure 1:**
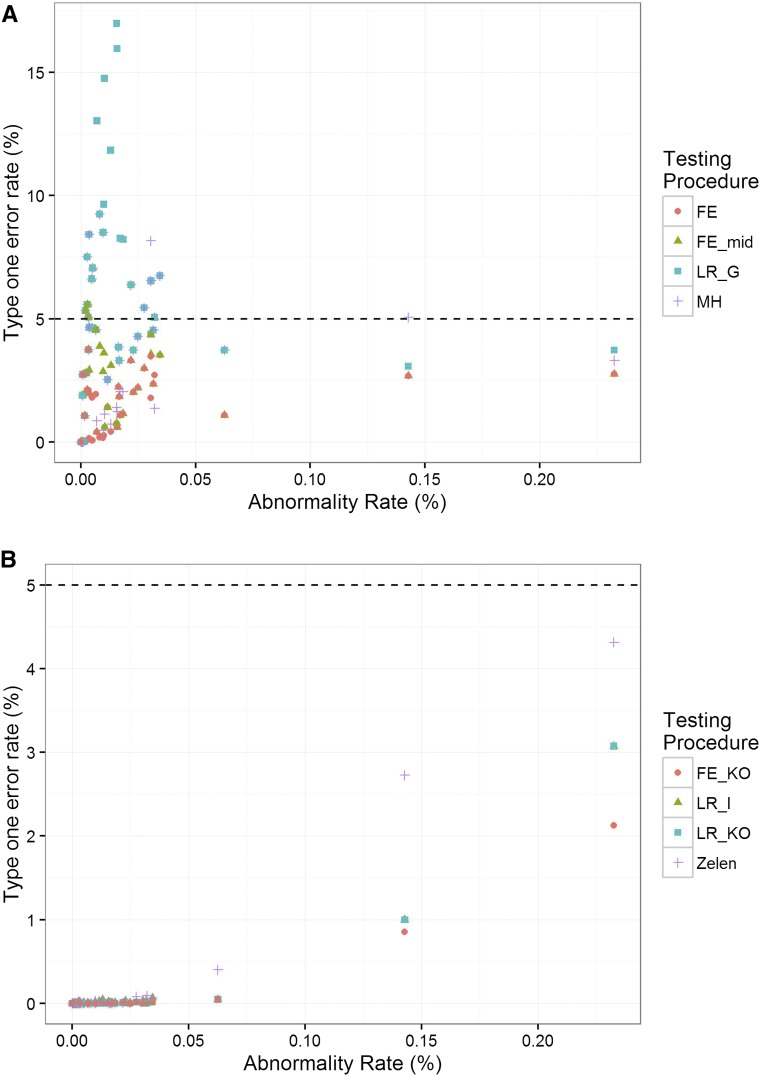
Resampling studies to assess the control of type-one errors. Resampling studies of control data to mirror a high-throughput phenotyping pipeline with 2 years of control data and seven knockout animals per sex were completed to assess the control of type-one error for the various methods at the 0.05 significance threshold. The type-one error rate from 15,000 × 100 (*i.e.*, number of iterations times number of variables) simulations is plotted as a function of the control data abnormality rate. (A) Stage 1 assessment of genotype effect. (B) Stage 2 assessment of genotype by sex interaction. The statistical methods compared are detailed in Table 1. FE, Fisher’s exact test; FE_KO, Fisher’s exact test mid-*P*-value using knockout data only; FE_mid, Fisher’s exact test mid-*P*-value; LR_G, biased reduction logistic regression; LR_I, biased reduction logistic regression with an interaction term; LR_KO, biased reduction logistic regression using knockout data only; MH, Mantel–Haenszel test; Zelen, Zelen mid-*P*-value.

In stage 1, testing the effect of genotype, all statistical methods were found to be conservative as the overall fraction of rejected hypotheses were below the nominal level when assessing the global behavior (across all traits and constructed data sets) (Figure S2). At the variable level, at times the type-one error rate was elevated for the LR_G and MH_mid approaches, and appeared to correlate with the abnormality rate in the wild-type data when the abnormality rate was between 0.00001 and 0.045% (Figure 1). The most extreme type-one error rates were observed in the LR_G implementation with a threefold higher error rate than expected. Similar results were seen at other significance thresholds (Figure S3) or when sampling real control data (Figure S4).

In stage 2, testing the interaction between the genotype and sex, the type-one error rate was highly conservative with no significant difference observed between the statistical methods (Figure S2). At the variable levels, the type-one error rate increased with abnormality rates; however, it remained conservative (Figure 1). Incorporating a filtering step, based on the outcome of the stage 1 testing, further reduced the type-one error rate. This is as expected, as significance in the second stage requires passing two tests (Figure S5). The impact of the various potential filters on the type-one error rate was also evaluated and their incorporation led to an increase in the type-one error rate that was closer, but still below, the nominal level (Figure S6).

**Figure 2:**
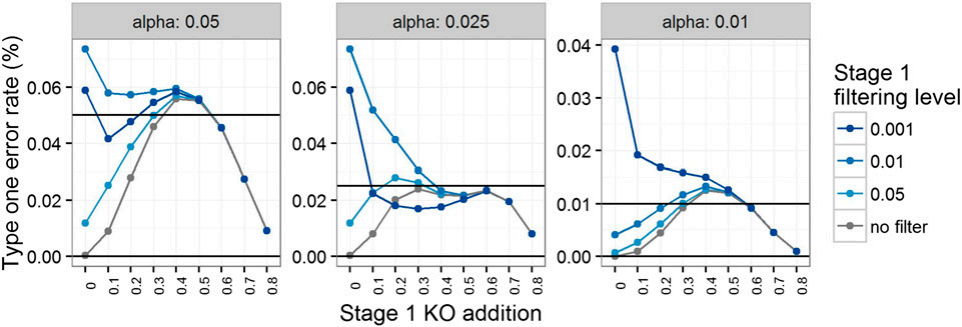
Simulations to study the type-one error rate for stage 2 in the presence of a genotype effect that affects both sexes equally. The type-one error rate (%) *vs.* the genotype effect of the LR_KO test for various significance thresholds following a main effect test using MH_mid at a fixed filtering level. Explored is the impact of a knockout addition (where abnormal signal was added to both male and female knockout mice) genotype effect and varying filter stringency between stage 1 and 2 for a variety of significance thresholds at stage 2 (α). The simulation is based on mimicking a high-throughput pipeline sampling seven male and seven female knockout animals and comparing them to a baseline data set (baseline abnormality rate of 0.05) collected over two years, assuming that seven male and seven female control animals were studied weekly. KO, knockout; LR_KO, biased reduction logistic regression using knockout data only; MH_mid, Mantel–Haenszel test mid-*P*-value.

The control of type-one errors at stage 2 was then assessed in the presence of a stage 1 genotype effect with and without filtering for significance at stage 1 (Figure 2). When the stage 1 genotype effect was large, filtering had little impact as the majority of genotype comparisons were significant at stage 1. However, when the genotype effect was small, filtering led to an inflation of the type-one error rate at stage 2, which at times went above nominal to some extent. We could not discern a consistent pattern in the impact of the filtering on the increase in type-one error rate, which we postulate is due to the discreteness of the *P*-value distribution.

### Assessing statistical power: sensitivity

**Figure 3:**
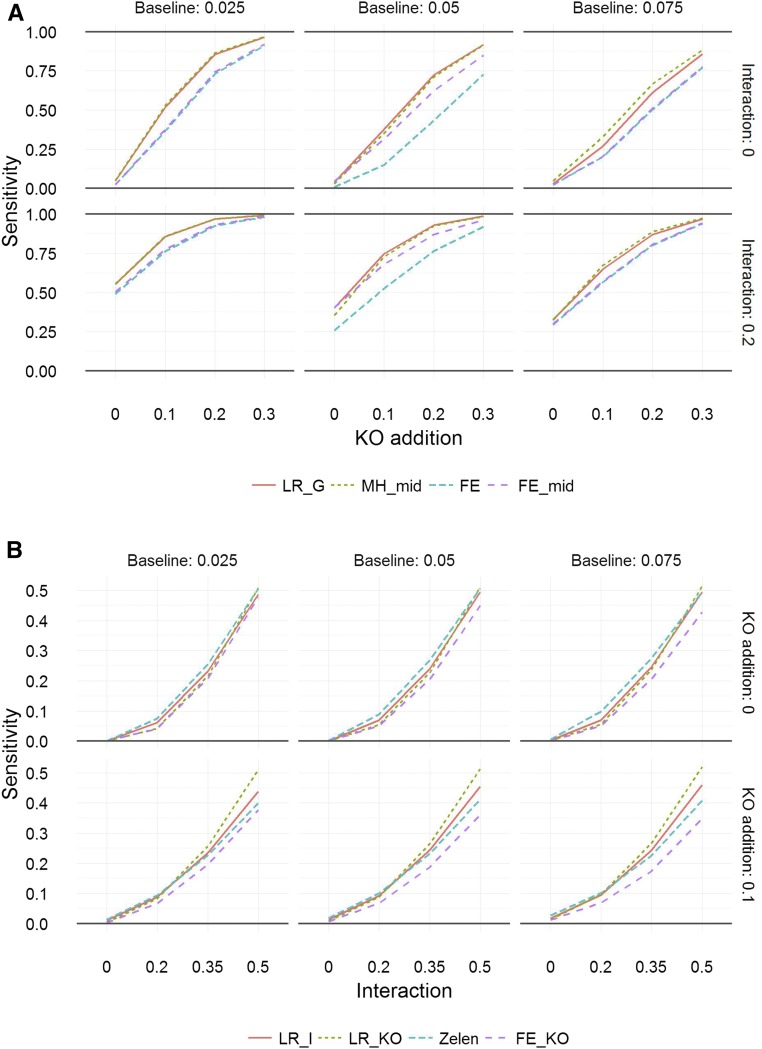
Sensitivity analysis from simulation studies. (A) Stage 1 power (the probability to detect genotype effect) *vs.* genotype effect for various baseline abnormality rates (in columns), where the abnormality rate is the same for both sexes in the wild-type mice, without interaction (top row) and with an additional effect for one of the sexes in the KO mice (lower row). (B) Stage 2 power (the probability to detect sexual dimorphism) *vs.* the additional effect for one of the sexes in the KO mice, for various baseline abnormality rates (in columns), without a main genotype effect (top row) and with a main genotype effect (bottom row). This is the assessment of sensitivity without any further workflow methodologies added. The simulations were based on sampling seven male and seven female knockout animals and comparing them to a baseline data set collected over two years, assuming that seven male and seven female control animals were studied weekly. The statistical methods compared are detailed in Table 1. FE, Fisher’s exact test; FE_KO, Fisher’s exact test mid-*P*-value for knockout data only; FE_mid, Fisher’s exact test mid-*P*-value; KO, knockout; LR_G, biased reduction logistic regression; LR_I, biased reduction logistic regression with an interaction term; LR_KO, biased reduction logistic regression using knockout data only; MH_mid, Mantel–Haenszel test mid-*P*-value; Zelen, Zelen mid-*P*-value.

A study was completed to assess the sensitivity of the various methods under simulations mirroring data obtained from a high-throughput pipeline after two years of operation. Focusing initially on stage 1 (Figure 3 and Figure S7), we find, unsurprisingly, that as the background abnormality rate increased the statistical power decreased. In the presence of an additional effect to one of the sexes (yielding an interaction effect) the power obviously increased, as the average rate further increased. When the background abnormality rate was zero, there were no differences in the statistical power between the four methods tested. Across all other scenarios, we can see the benefits of the mid-*P*-value increasing power compared to the traditional FE. The power was highest for the statistical tests that aggregate over the two sexes (LR_G and MH_mid). In summary, for the majority of scenarios, we find that the power was very similar between LR_G and MH_mid, followed by the FE_mid, with the traditional statistical method (FE) having the lowest power. The power was similar between the LR_G and MH_mid methods; however, the LR_G approach sometimes failed to control the type-one error and therefore we selected the MH_mid test in what follows.

For the second stage testing (Figure 3 and Figure S8), when the abnormality rate in the wild-type was zero, the Zelen and LR_I tests had no power to detect an interaction. The zero power arose because these tests include both the wild-type and knockout data and are conditioning on the margins. This is a significant issue for their use as depending on the data set size there will frequently be no abnormalities observed in the wild-type data. At an abnormality rate of zero for the wild-type mice, the LR_KO approach had greater sensitivity than the FE_KO approach. At abnormality rates above zero for the wild-type mice, in the absence of a main effect, the power was very similar between methods. In the presence of a main effect, LR_KO power was bigger than LR_I power, which in turn was bigger than Zelen power and finally bigger than the power of FE_KO. In summary, we can conclude that the LR_KO method was most sensitive. The strategy of filtering based on the stage 1 testing of the genotype effect was found to have little impact on the sensitivity of stage 2 (data not shown). As all statistical tests from the studies assessing the reliability of call had similar behavior, the LR_KO method was selected as the test to proceed with due to the higher sensitivity.

### Results from applying various multiple testing methodologies

**Figure 4:**
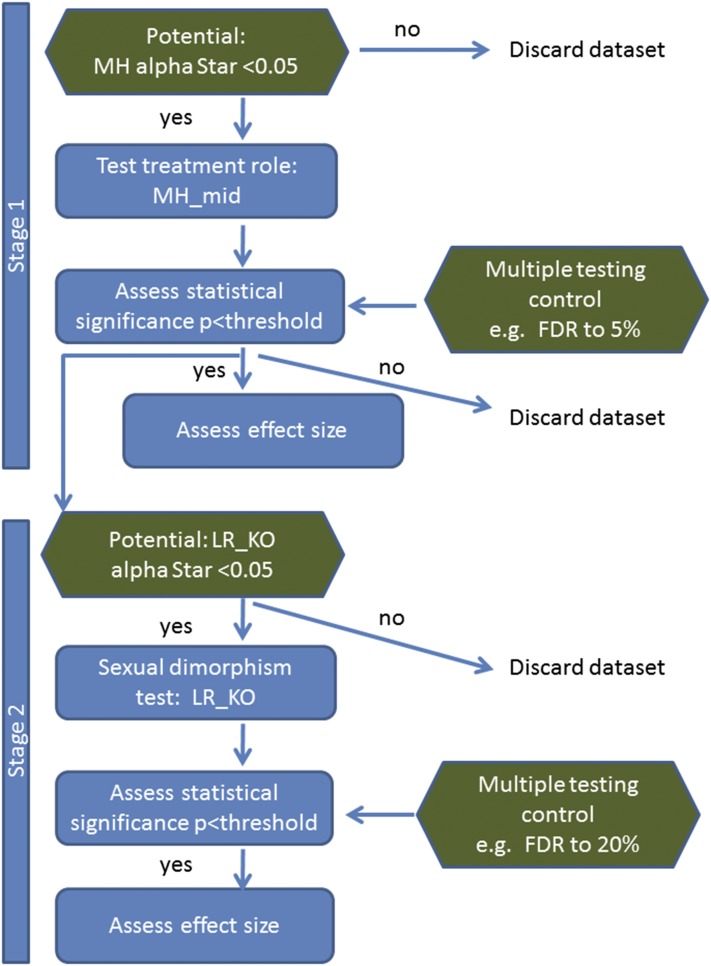
Proposed analysis pipeline. Flow diagram summarizing the proposed methodology for a two-stage process assessing for a treatment and a treatment by sex interaction for rare event categorical data. Shown in green are steps included to manage the multiple testing problem common with phenotyping studies as many traits are monitored. In the case study example, the treatment is genotype; however, other treatments could be compared (*e.g.*, diet). The steps where data sets are discarded arise as the corresponding hypotheses cannot reach significance even with the most extreme result, and hence are not included in further statistical analysis. FDR, false discovery rate; LR_KO, biased reduction logistic regression using knockout data only; MH α star, Mantel–Haenszel test α star; MH_mid, Mantel–Haenszel test mid-*P*-value.

The statistical tests that were identified as valid and powerful, namely MH_mid for stage 1 and LR_KO for stage 2 (Figure 4), were applied to data from the high-throughput phenotyping pipeline at WTSI. The data comprised 99,530 wild-type–knockout data sets arising from 580 families (gene knockouts and associated zygosity), characterized with up to three phenotyping screens (with 26–182 traits per knockout line). We have incorporated a number of strategies to manage the multiple testing problem prevalent in phenotyping studies. Filtering based on the α star, to remove data sets that did not have the potential for significant hypotheses, reduced the multiple testing burden substantially. We considered a number of approaches that manage the FDR. The number of calls made from a variety of selection approaches is shown in Table 2. The conservative nature of the IMPC threshold (α = 0.0001) gave the lowest number of significant calls for stage 1 (*n* = 268). At stage 2, no significant sex dimorphism calls were made, which is as expected since even with complete penetrance with seven knockouts mice per sex the *P*-value obtained cannot be smaller than the IMPC threshold for significance (Table 3). Filtering at stage 1, thereby pursuing only the tests that have the potential to reach below the 5% level with the MH_mid test (*i.e.*, MH_mid α star < 5%), reduced the number of hypotheses tested by 46.6%. Adding the stage 2 potential filter, LR_KO α star, to those significant at stage 1 further reduced the number of hypotheses tested by 65.6%. The reduction in the number of hypotheses by the inclusion of the filtering step increased sensitivity by increasing in the number of calls made at stage 1 by 19% (approach 1 *vs.* approach 3 Table 2).

**Table 2:** Comparison of significant calls made by various adaptive methods for managing the multiple testing burden

Approach	1	2	3	4	5	6	7	8	9	10
MH α star filter applied?		Yes			Yes	Yes			Yes	Yes
Amend the FDR control to be within family?							Yes	Yes	Yes	Yes
Number of calls stage 1	298	355	298	298	355	355	301	301	301	301
Apply filter for significant at stage 1?			Yes	Yes	Yes	Yes	Yes	Yes	Yes	Yes
Apply LR_KO α star filter?		Yes							Yes	Yes
Amend the FDR control to be within family?							Yes	Yes	Yes	Yes
FDR threshold (in %) applied at stage 2	5	5	5	20	5	20	5	20	5	20
Number of calls stage 2	0	0	0	37	0	48	0	21	0	36

Comparison of the number of significant calls made with the two-step analysis pipeline when using a variety of methods to manage the multiple testing problem for the Wellcome Trust Sanger Institute wild-type–knockout data sets (*n* = 99,530) from 580 knockout families (gene and zygosity) studied with up to three screens (number of traits per line 26–182). The approaches differ (as indicated by the Yes statement) in the filters applied, whether the procedure used is aimed at controlling the FDR across the whole data set or controlled on average across the families, and the significance threshold used at stage 2. For stage 1, testing of genotype effect, a 5% FDR threshold was applied. MH α star, Mantel–Haenszel test α star; FDR, false discovery rate; LR_KO, biased reduction logistic regression using knockout data only.

**Table 3:** The possible *P*-values obtainable for stage 2 test of genotype by sex interaction

Penetrance (%)	*n*/Sex	Number Abnormalities in One Sex	Minimum Obtainable *P*-Value (LR_KO α Star)
14.2	7	1	0.44059
28.6	7	2	0.18407
42.9	7	3	0.06970
57.1	7	4	0.02299
71.4	7	5	0.00620
85.7	7	6	0.00121
100	7	7	0.00013

The minimum obtainable *P*-value, α star, from the stage 2 testing for sexual dimorphism within data sets that consist of seven knockout male and seven knockout female mice, with varying amounts of observed abnormalities in one of the sexes. The stage 2 test of sexual dimorphism was the LR_KO test comparing the abnormal proportions between the male and female data. LR_KO, biased reduction logistic regression using knockout data only.

**Figure 5:**
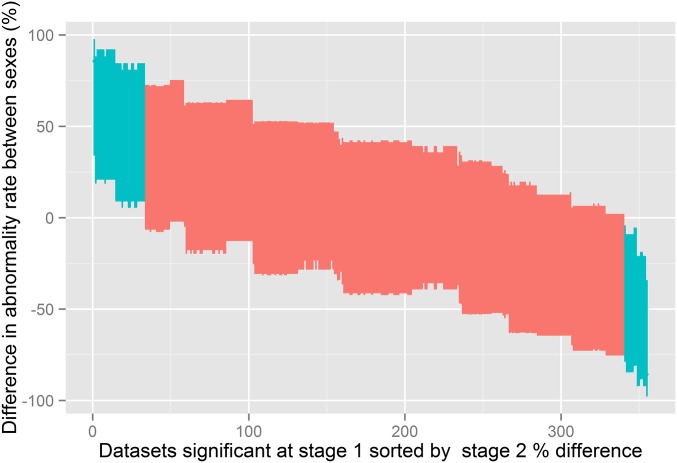
The sexual dimorphism effect size measure. The 95% Newcombe confidence interval for the difference in abnormality rate between the male and female knockout mice for the Wellcome Trust Sanger Institute data sets of hypotheses selected as significant at stage 1 (*n* = 355) (approach six in Table 2). C.I. are sorted by the estimated percentage change in abnormality rate between the sexes in the knockout mice. Those classed as sexually dimorphic at stage 2 (*n* = 48) using a 20% false discovery rate (approach 6 in Table 2) are colored jade. No adjustment to the C.I. for multiple testing has been included as all C.I. are presented.

When applying the FDR-controlling procedure at the 0.2 level for stage 2 (approach 6 in Table 2), 48 wild-type–knockout data sets would be classed as sexually dimorphic and, with the 20% FDR, we would expect that 10 of these are false calls. When applying the FDR-controlling procedure at the 0.05 level for stage 2, none of the stage 2 hypotheses were classed as significant (approach 5, 7, and 9 in Table 2). This is unsurprising as with only six mice per sex there is low power to detect a difference in proportion. Furthermore, the minimum *P*-value possible with complete penetrance of a sexually dimorphic phenotype is 0.00013, while with 72% penetrance the minimum *P*-value would be 0.0062 (Table 3). The impact of sex on the genotype effect can be assessed by constructing 95% C.I. for the difference in abnormality rate (Figure 5), those classed as having a statistical interaction between genotype and sex had an estimated difference in abnormality rate between the sexes > 50%. For researchers interested in the effect size of only genes with a proven genotype*sex interaction, the C.I. should be further inflated because when only a subset of C.I. are selected (for presentation or reporting) there is a need to adjust the C.I. for the selection (Benjamini and Yekutieli 2005). However, with the high-throughput case study example here, the high multiplicity, low sample size of the study, and the low frequency of phenotypes means that the adjusted C.I. would be very wide.

An alternative strategy is to control the average FDR across families using the BB method, where a family is defined by grouping data by gene and zygosity. An advantage of the BB method is that it is hierarchical and hence you can first screen the families for which a detailed analysis is desired while controlling the FDR of gene families, and then test within the selected families to control an average error rate. We found that sensitivity was decreased at both stage 1 and stage 2 in this data set compared to the BH method with its global control over genes and phenotypes (approach 7 and 9 in Table 2). We perceive the method did not improve sensitivity because in this data set, with the rare nature of phenotypic abnormalities, there was a lack of clustering to improve the sensitivity compared to global control.

### Overview of the biological findings

Three screens (eye dysmorphology, NAMPA, and skeletal) performed on 580 alleles identified 355 significant hits, of which 13.5% were classed as sexually dimorphic. The low hit rate (0.4% of the data sets generated) is as expected for the traits studied. The analysis achieved the hypothesis-generating objective of the project, and since the literature is currently very sparse for studies considering the role of sex in phenotypic expression, this limited our ability to validate the findings. Consider the gene with the highest number of abnormal traits (21), homozygous *Myo10^tm2(KOMP)Wtsi^* (MGI:107716), which codes for an actin-based motor protein. The abnormalities clustered around issues with the eye including the lens, cornea, and iris, and around the paw morphology. While the hindpaw abnormalities had a similar abnormality rate in the sexes (females 43% abnormal and males 43% abnormal, BH-adjusted *P*-value = 7.08e−23), the forepaw showed a greater penetrance of the abnormality for the females for the forepaw digit fusion (females 100% abnormal while males 43% abnormality stage 2 BH-adjusted *P*-value = 0.1333). This gene has also been studied as a spontaneous mutation *Myo10^m1J^/Myo10^m1J^* (MGI:5578506) and similar sexually dimorphic abnormalities were identified (Karst *et al.* 2014). Further validation and exploration work will build on this knowledge.

### Conclusions

Functional genetic studies typically aim to infer on a battery of phenotypes, many of which are discrete and rare. There are multiple challenges to making robust calls; those arising from the rare events nature of the data, the multiple testing issue arising from monitoring many traits, and the need to assess for both a treatment effect and an interaction between treatment and sex effect. We have developed and demonstrated a powerful two-step process while offering false positive rate control. This manuscript focuses on the statistical development needed; a second manuscript applying the developed approach across the IMPC data will be submitted shortly (Natasha A. Karp, Jeremy Mason, Arthur L. Beaudet, Yoav Benjamini, Lynette Bower, Robert E. Braun, Steve Brown, Elissa J. Chesler, Mary E. Dickinson, Ann M. Flenniken, Helmut Fuchs^0^, Xiang Gao, Shiying Guo, Simon Greenaway, Ruth Heller, Yann Herault, Martin Hrabe de Angelis, Monica J. Justice, Natalja Kurbatova, Christopher J. Lelliott, K.C. Kent Lloyd, Ann-Marie Mallon, Judith E. Mank, Hiroshi Masuya, Colin McKerlie, Terrence F. Meehan, Richard F. Mott, Stephen A. Murray, Helen Parkinson, Ramiro Ramirez-Solis, Luis Santos, John R. Seavitt, Damian Smedley, Tania Sorg, Anneliese O. Speak, Karen Steel, Karen L. Svenson, The International Mouse Phenotyping Consortium, Shigeharu Wakana, David West, Sara Wells, Henrik Westerberg, Shay Yaacoby, Jacqueline K. White, unpublished results). File S3 and File S4 demonstrate how these methods can be run on a data set.

To date, the IMPC community has utilized a fixed threshold (*P*-value < 0.0001) in the automated annotation pipeline (Koscielny *et al.* 2013). In this data set, we find that this fixed threshold for stage 1 was a conservative method and returned calls with a FDR < 5%. Moreover, for stage 2, a sexual dimorphic call would never be made as the maximal obtainable *P*-value would never exceed the fixed threshold with a design of seven male and seven female knockout mice.

The pipeline developed here assesses the genotype effect at stage 1 using a Mantel–Haenszel test with mid-*P* modification, which tests for a difference in proportions by treatment for both sexes, and tests the sexual dimorphism at stage 2 only in data sets where the genotype effect was significant at stage 1. The test for sexual dimorphism is conducted in the knockout mice only using Firth’s bias reduced likelihood ratio. At both stages, we further relied on filtering those data sets where significance cannot be achieved even under the most extreme results. The advantage of utilizing this approach, summarized in Figure 4, was demonstrated by an increased sensitivity at both stages.

The analysis implemented within this manuscript looks at methods for controlling the FDR in a single combined analysis of all knockout lines in the data set. The IMPC analysis pipeline on the portal has the additional challenge that the data set is continually growing and would benefit from making statistical calls on knockout data once all phenotyping for a particular knockout line is completed. Moving forward, research is needed into methodologies that can respond to the preceding calls with online FDR control, see (Aharoni and Saharon 2014; Javanmard and Montanari 2016) and the references within.

## 

## Supplementary Material

Supplemental material is available online at www.genetics.org/lookup/suppl/doi:10.1534/genetics.116.195388/-/DC1.
