# Supplementary figures and images for: Improving the Identification of Phenotypic Abnormalities and Sexual Dimorphism in Mice When Studying Rare Event Categorical Characteristics
Source: Genetics. 2016 Dec 5;205(2):491–501. doi: 10.1534/genetics.116.195388 (PMC5289831; doi:10.1534/genetics.116.195388)

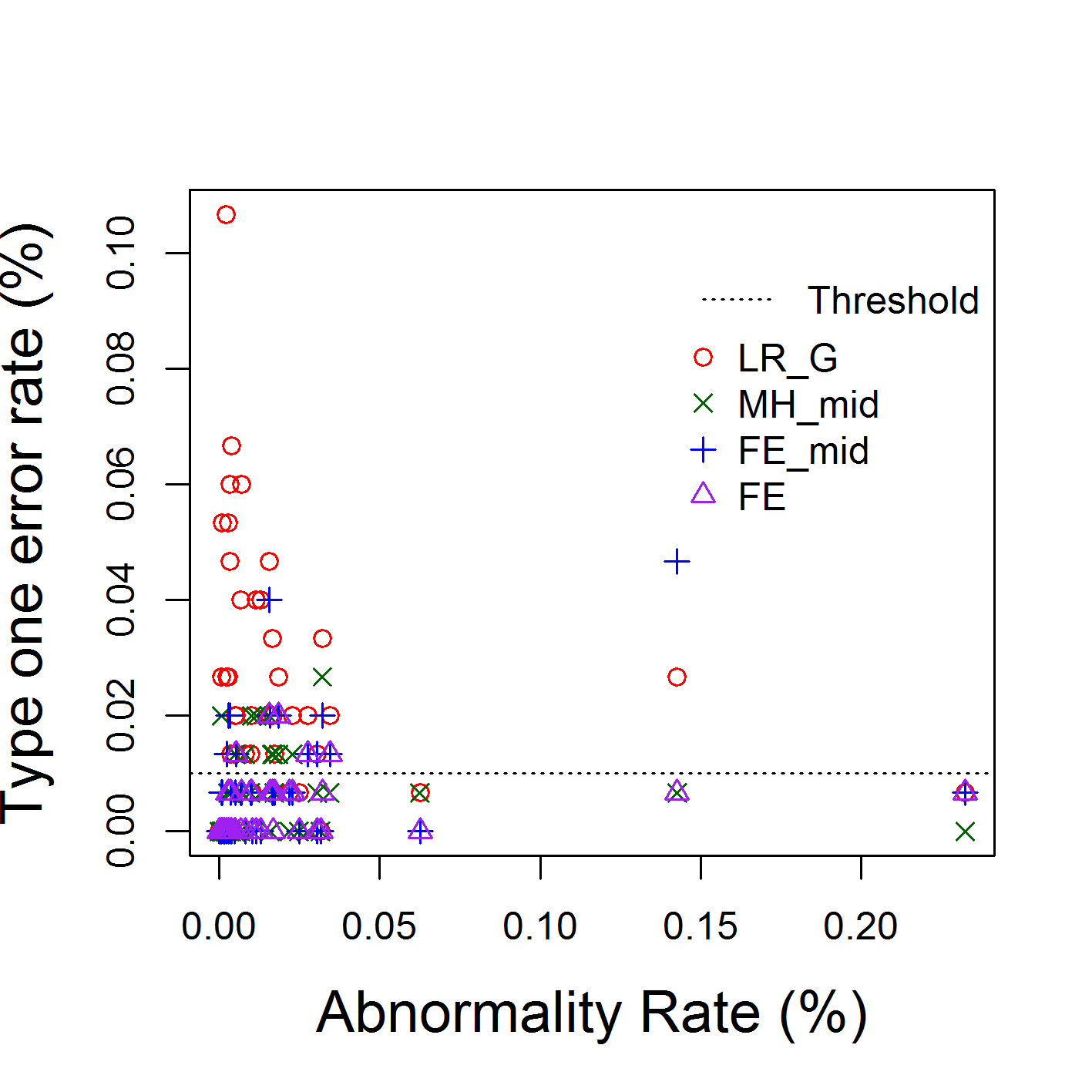

Supplement: Supplementary file 3 [file 491FigureS3.tiff]

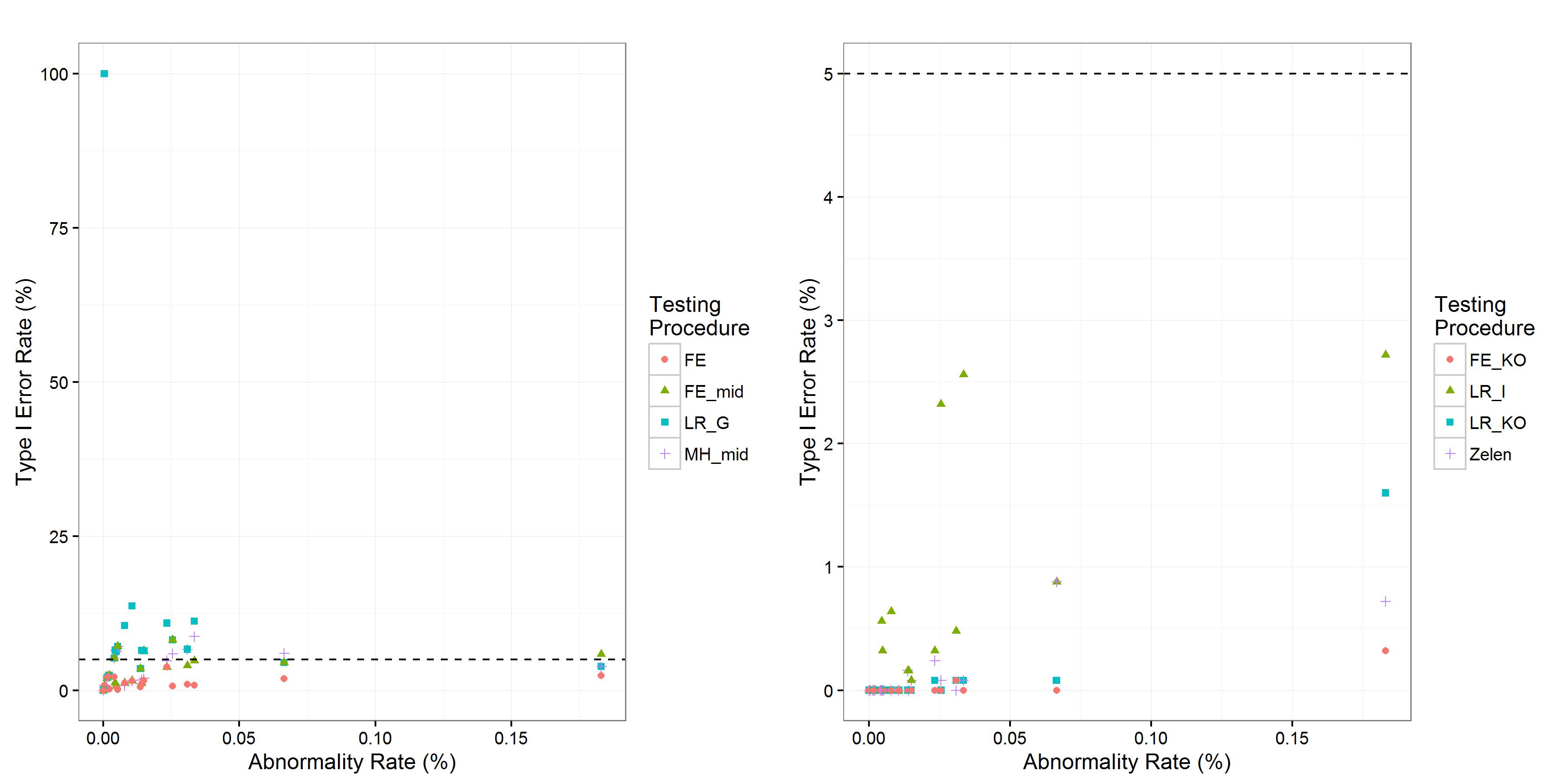

Supplement: Supplementary file 4 [file 491FigureS4.jpeg]

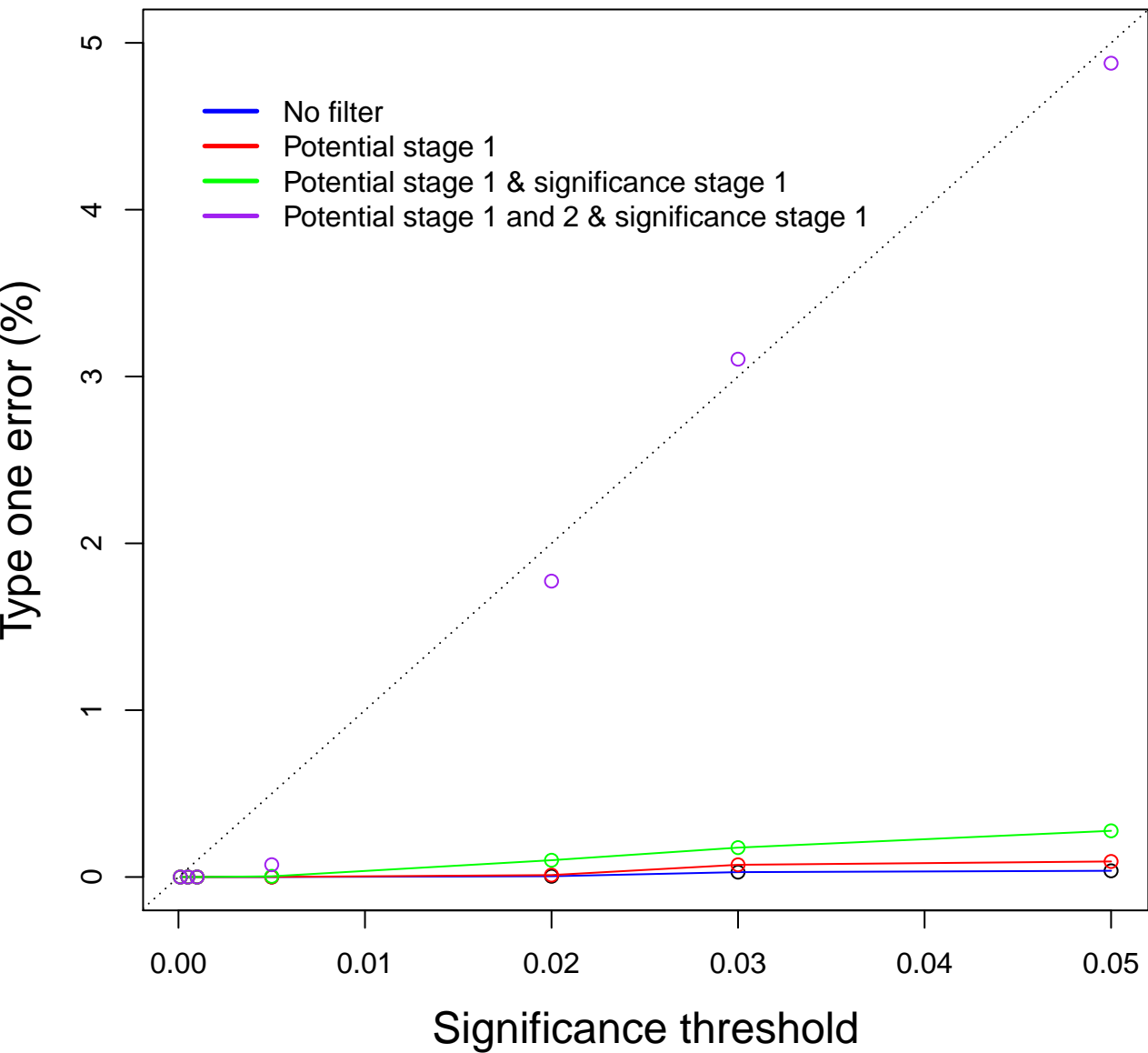

Supplement: Supplementary file 6 [file 491FigureS6.pdf]

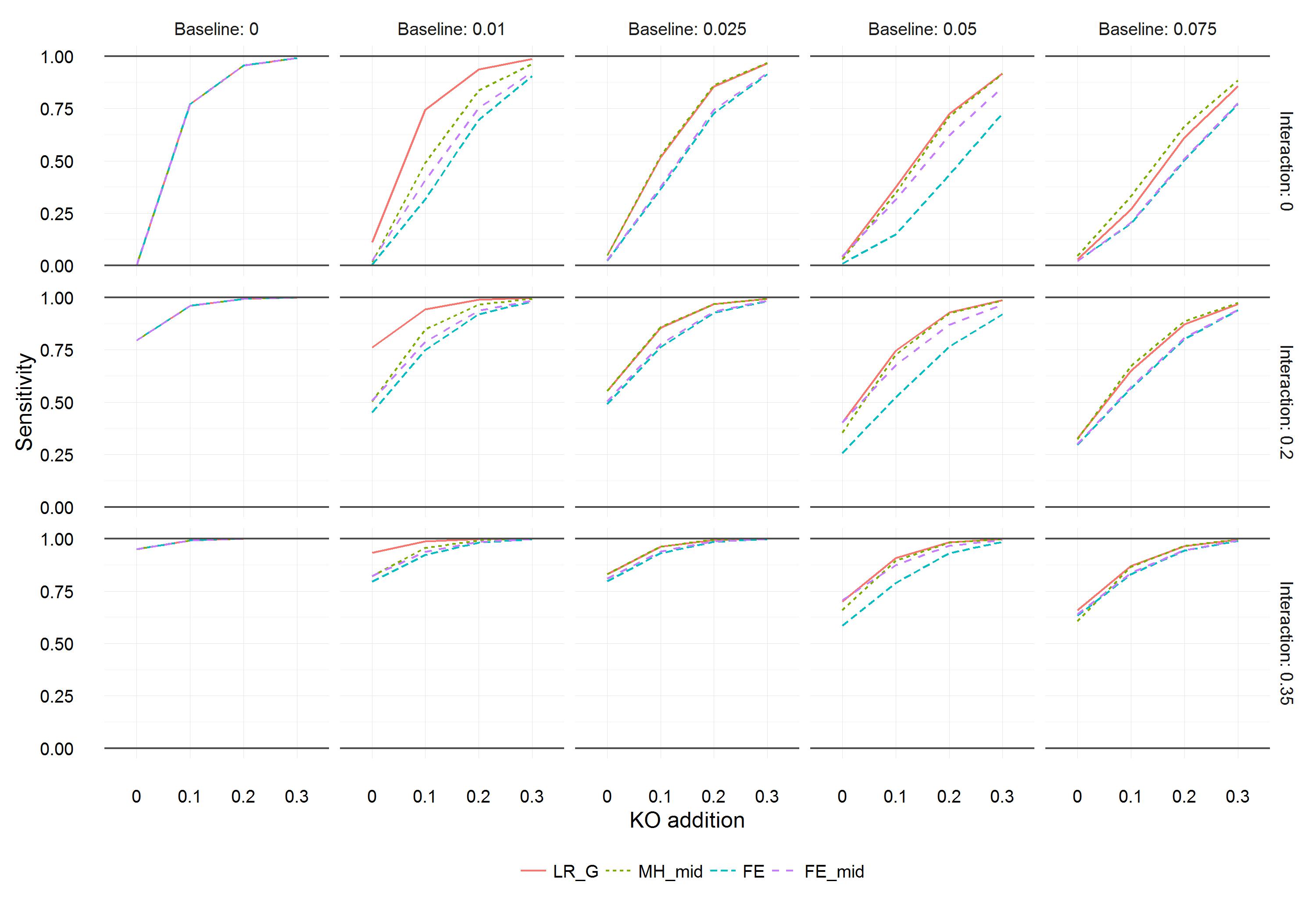

Supplement: Supplementary file 7 [file 491FigureS7.jpg]

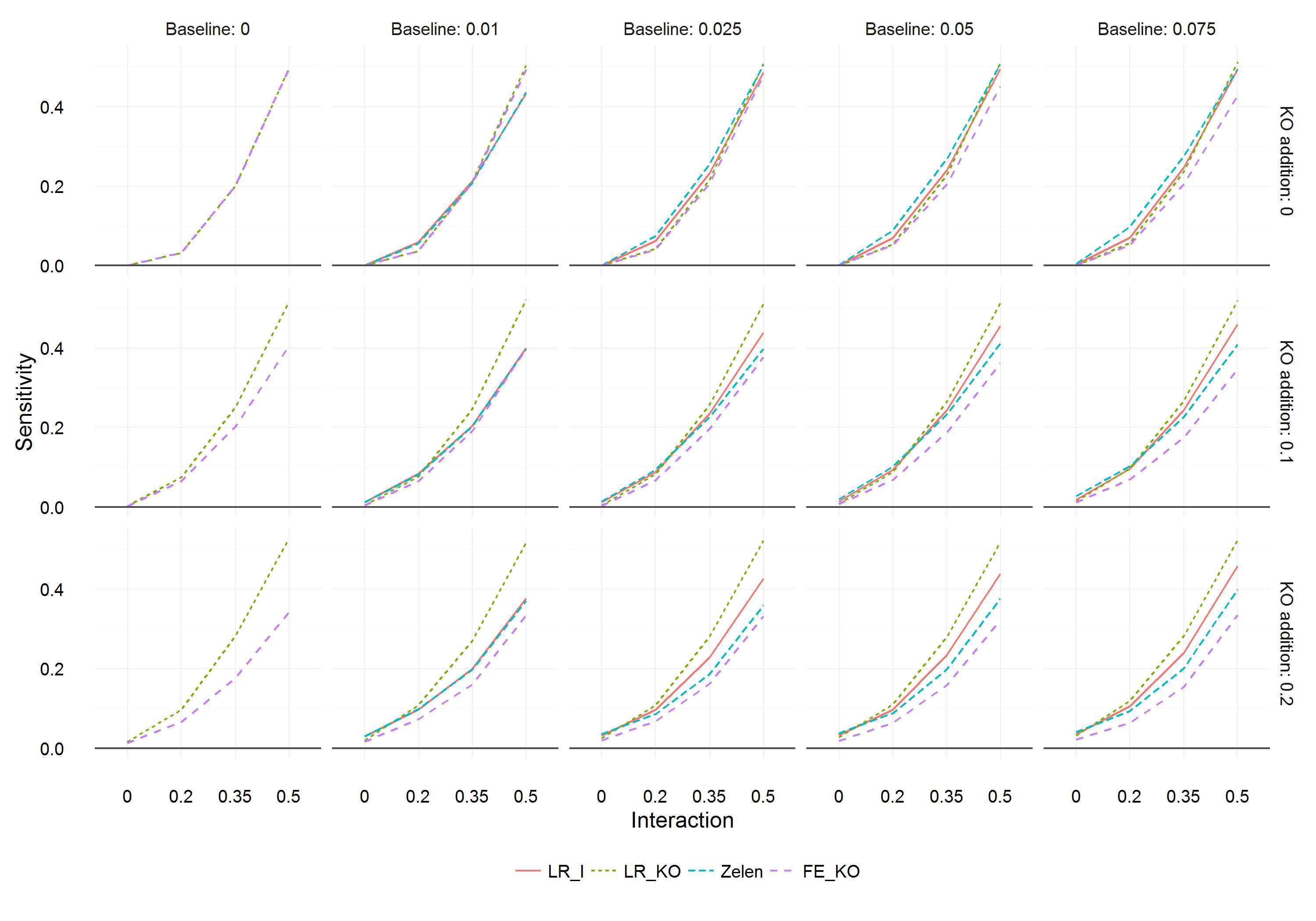

Supplement: Supplementary file 8 [file 491FigureS8.jpg]
