# Supplementary material for: Improving the Identification of Phenotypic Abnormalities and Sexual Dimorphism in Mice When Studying Rare Event Categorical Characteristics
Source: Genetics. 2016 Dec 5;205(2):491–501. doi: 10.1534/genetics.116.195388 (PMC5289831; doi:10.1534/genetics.116.195388)
Supplement: Supplementary file 9 [file 491FileS1.docx]

## Supplementary Methods 1: Data production

## Ethics Statement:

The care and use of mice in the Wellcome Trust Sanger Institute (WTSI) study was carried out in accordance with UK Home Office regulations, UK Animals (Scientific Procedures) Act of 1986 under two UK Home Office licenses which approved this work (80/2076 and 80/2485) which were reviewed regularly by the WTSI Animal Welfare and Ethical Review Board. Animal welfare was assessed routinely for all mice involved. Adult mice were killed by terminal anesthesia followed by exsanguination and either cervical dislocation or removal of the heart.

## Mouse generation, genotype and allele quality control

Mice carrying knockout first conditional-ready alleles were generated as described previously ([White *et al.* 2013](#_ENREF_34)). Embryonic stem cell quality control was performed as described ([Skarnes *et al.* 2011](#_ENREF_32)). Furthermore, molecular characterization of mutant mouse strains was performed as described previously ([Ryder *et al.* 2013](#_ENREF_31)). Upon completion of phenotyping, genotyping was repeated and data were only accepted from mice for which the second genotype was concordant with the original genotype.

## Housing and husbandry

Mice were maintained in a specific pathogen free unit on a 12hr light: 12hr dark cycle with lights off at 7:30pm and no twilight period. The ambient temperature was 21 ± 2^o^C and the humidity was 55 ± 10%. Mice were housed for phenotyping using a stocking density of 3-5 mice per cage (overall dimensions of caging: (L x W x H) 365 x 207 x 140mm, floor area 530cm^2^) in individually ventilated caging (Techniplast Seal Safe1284L) receiving 60 air changes per hour. In addition to Aspen bedding substrate, standard environmental enrichment of two nestlets, a cardboard Fun Tunnel and three wooden chew blocks were provided. Mice were given water and diet *ad libitum,* unless otherwise stated. Mice studied in the ‘MGP Select’ pipeline were maintained on Mouse Breeders Diet (Lab Diets, 5021-3) throughout their lifetime. At 4 weeks of age, mice on the ‘Mouse GP’ pipeline were transferred from Mouse Breeders Diet (Lab Diets, 5021-3) to a high fat (21.4% fat by crude content; 42% calories provided by fat) dietary challenge (Special Diet Services, Western RD 829100).

## Phenotypic data

The analysis uses data taken from high-throughput phenotyping, which is based on a pipeline concept where a mouse is characterized by a series of standardized and validated set of tests underpinned by standard operating procedures. The phenotyping tests chosen cover a variety of disease-related and biological systems, including the metabolic, cardiovascular, bone, neurological and behavioral, sensory and hematological systems and clinical chemistry. The data were obtained as detailed in ([White *et al.* 2013](#_ENREF_34)) following the standard operating procedures at IMPReSS ([www.mousephenotype.org/impress](http://www.mousephenotype.org/impress)). Factors thought to affect the variables were standardized as far as possible. Where standardization was not possible, steps were taken to reduce potential bias. For example, at WTSI the impact of different people completing the experiment was minimized (“minimized operator”) as defined in the [Mouse Experimental Design Ontology](http://bioportal.bioontology.org/ontologies/MEDO) (MEDO) ([Karp *et al.* 2015](#_ENREF_18)) as “The process by which steps are taken to minimize the potential differences in the effector by training and monitoring of operator.” (<http://bioportal.bioontology.org/ontologies/MEDO/?p=summary>). The data captured with the MEDO ontology can be accessed at http://www.mousephenotype.org/about-impc/arrive-guidelines.

##

## Pre-set reasons are established for QC failures (e.g. insufficient sample) and detailed within IMPRESS ([www.mousephenotype.org/impress](http://www.mousephenotype.org/impress)) providing standardized options as agreed by area experts as to when data can be discarded. Data can only be QC failed from the dataset if clear technical reasons can be found for a measurement being an outlier. Reasons are provided and this is tracked within the database.

## Experimental design

Phenotyping data are collected at regular intervals on age-matched wildtype (control) mice of equivalent genetic backgrounds. Cohorts of at least seven homozygote mice of each sex per knockout line were generated. If no homozygotes were obtained from 28 or more offspring of heterozygote intercrosses, the line was deemed homozygous lethal. Similarly, if less than 13% of the pups resulting from intercrossing were homozygous, the line was judged as being homozygous subviable. In such circumstances, heterozygote mice were committed to the phenotyping pipelines.

The random allocation of mice to experimental group (wildtype versus knockout) was driven by Mendelian Inheritance. Reflecting the high-throughput nature of the phenotyping pipeline, blinding to the identity of knockout lines during phenotyping was not employed as the cage cards include genotype information, though with a high throughput environment without a defined hypothesis, the potential bias is minimized. The individual mouse was considered the experimental unit within the studies. Further detailed experimental design information (e.g. exact definition of a control animal) is captured by with a standardized ontology as detailed in Karp *et al* ([Karp *et al.* 2015](#_ENREF_18)) and is available from the IMPC portal (http://www.mousephenotype.org/about-impc/arrive-guidelines).

## Datasets

Wildtype-knockout datasets: Data was extracted from the WTSI database for the ‘MGP Select’ pipeline on genetic background C57BL/6N (MGI:2159965) and three categorical screens: Skeletal (extraction date: 29/10/2014), Eye (extraction date: 11/6/15) and Neurological and morphology phenotypic assessment (NAMPA) (extraction date: 11/6/15). For each variable of interest, the data were recoded to 0 to represent “as expected phenotypes” and 1 to represent “not as expected phenotypes”. Knockout-wildtype datasets were only processed if there were 7 or more knockout mice per sex. Table 1 summaries the number of mice, number of lines and traits monitored within these datasets. Datasets could be grouped by family which was classed as datasets arising for a knockout line for a zygosity (heterozygous or homozygous).

Wildtype data for exploration: Data was extracted from the WTSI database for the ‘Mouse GP’ pipeline on genetic background C57BL/6N (MGI:2159965) for three categorical screens: skeletal, eye and neurological and morphology phenotypic assessment (NAMPA). The pipeline ran from August 2009 until February 2012 and collected wildtype data with seven males and females weekly (Table 2).

## Table 1: Control-knockout dataset characteristics

Characteristics of wildtype-knockout datasets extracted from the WTSI MGP Select Pipeline.

| Assay | No. wildtype male mice | No.  wildtype  female  mice | Max. male knockout mice | Max. female knockout mice per dataset | Number traits monitored |
| --- | --- | --- | --- | --- | --- |
| Eye | 1134 | 1142 | 14 | 10 | 26 |
| Skeletal | 1103 | 1106 | 14 | 10 | 42 |
| NAMPA | 1378 | 1385 | 14 | 14 | 114 |

## Table 2: Control dataset characteristics

Characteristics of wildtype datasets extracted from the WTSI Mouse GP Pipeline.

| Assay | Assay dates | Number mice | Number traits monitored |
| --- | --- | --- | --- |
| Eye | 348 | 2276 | 26 |
| Skeletal | 503 | 2763 | 42 |
| NAMPA | 466 | 2209 | 114 |

## 
