# Supplementary material for: Improving the Identification of Phenotypic Abnormalities and Sexual Dimorphism in Mice When Studying Rare Event Categorical Characteristics
Source: Genetics. 2016 Dec 5;205(2):491–501. doi: 10.1534/genetics.116.195388 (PMC5289831; doi:10.1534/genetics.116.195388)
Supplement: Supplementary file 10 [file 491FileS2.docx]

S1 Methods: Mid-p-value calculations in The Discrete Testing Procedures

We focus on the experimental results of abnormalities counts, of *one* variable, in males and females groups of *one* Knockout (KO) and in the males and females Wildtype (WT) groups.

We summaries these results in a 2X2X2 contingency table with the notations as follows:

|  |  | KO | WT | Totals |
| --- | --- | --- | --- | --- |
| Abnormality | Yes |  |  | $n_{1.i}$ |
|  | No |  |  | $n_{2.i}$ |
|  | Totals | $n_{.1i}$ | $n_{.2i}$ | $n_{..i}$ |

where $i=M,F$ denote the stratum of the males or females groups.

### Stage one tests

#### Two Fisher Exact Tests

In each sex stratum, we perform Fisher Exact (FE) test to test whether the abnormality rate in the KO groups is higher than in the WT groups.

The test is one-sided, testing for significance of higher rate in the *KO* groups.

Under the null hypothesis the test statistic has hyper-geometrical distribution:

The p-value and mid-p-value for an observed test statistic value are:

where $n_{Ui}=min(n_{1.i},n_{.1i})$.

Aggregating over both males, and females the p-value and mid p-value for the null hypothesis of no genotype effect is $2\min(P_{M},P_{F})$ and $2\min(midP_{M},midP_{F})$ respectively.

#### Exact Mantel-Haenszel test

The Mantel-Haenszel (MH) Test tests for the genotype effect on the abnormality rate, while controlling for the sex variable effect.

Using the notations as above, the MH test statistic is

The distribution of the statistic is the convolution of the two hyper-geometrical distributions of each sex

$$f_{MH}=f_{F}*f_{M}$$

The null distribution is conditional on same the margin totals as in the two FE tests: $n_{1.M},n_{.1M},n_{1.F},n_{.1F}$.

The p-value and mid-p-value are defined similarly

where $n_{U}=n_{UM}+n_{UF}$.

### Stage two tests

#### Fisher Exact Test on the KO groups only

Here we apply **two-sided** FE test on the KO groups only (males and females). The test assess for the sex effect on the abnormality rate within the KO stratum.

Since the analysis focus on the KO groups, we look at the table of this stratum only:

|  |  | Males | Females | Totals |
| --- | --- | --- | --- | --- |
| Abnormality | Yes |  |  | $n'_{1.}$ |
|  | No |  |  | $n'_{2.}$ |
|  | Totals | $n'_{.1}$ | $n'_{.2}$ | $n'_{..}$ |

(Here correspond to , correspond to , $n'_{1.}$ correspond to their sum - $n_{11.}$,...)

The test statistic has hyper-geometrical distribution:

The p-value and mid-p-value for an observed test statistic value are:

#### Zelen test for a Common Odds Ratio

With the Zelen test we assess the sexual dimorphism manifested as interaction between the genotype and the sex effects. The Zelen test is an exact test. Its null hypothesis is whether the odds ratios are common in both sex levels.

The null distribution is conditional on , as well as the margins $n_{.1F},n_{1.F},n_{..F},n_{.1M},n_{1.M},n_{..M}$.

Then the p-value and mid-p-value are

.

References

Agresti A, Kateri M. 2011. *Categorical data analysis*. Springer Berlin Heidelberg.

Hollander M, Wolfe DA, Chicken, E. 2013. *Nonparametric statistical methods*. John Wiley & Sons.
