# Supplementary material for: Improving the Identification of Phenotypic Abnormalities and Sexual Dimorphism in Mice When Studying Rare Event Categorical Characteristics
Source: Genetics. 2016 Dec 5;205(2):491–501. doi: 10.1534/genetics.116.195388 (PMC5289831; doi:10.1534/genetics.116.195388)
Supplement: Supplementary file 12 [file 491FileS3.docx]

File S3. An example dataset. (.csv, 881 KB)

[www.genetics.org/lookup/suppl/doi:10.1534/genetics.116.195388/-/DC1/FileS3.csv](http://www.genetics.org/lookup/suppl/doi:10.1534/genetics.116.195388/-/DC1/FileS3.csv)
