# Supplementary material for: Improving the Identification of Phenotypic Abnormalities and Sexual Dimorphism in Mice When Studying Rare Event Categorical Characteristics
Source: Genetics. 2016 Dec 5;205(2):491–501. doi: 10.1534/genetics.116.195388 (PMC5289831; doi:10.1534/genetics.116.195388)
Supplement: Supplementary file 13 [file 491FileS4.docx]

RecommendedTwoStepProcess - worked example

Natasha Karp

12 October 2016

## Overview

This document demonstrates the recommended methodology for a dataset.

- First we input the data for a gene and a screen.
- Then we input the various functions needed to run the analysis
- In the next section, we demostrate for a single variable the generation of the statistical output.
- This is followed by a section which loops around and collects the statistical output for many variables for the dataset.
- In the final section, using the preceding output, we demonstrate the management of multiple testing using a mixture of adjusting for multiple testing but only after selecting for potential.

## Inputting the data for analysis

The analysis requires a dataframe with one row for each animal. The analysis requires the following columns * Genotype - with two strings to represent the testGenotype and refGenotype (e.g. WT and HOM) * Sex - with two strings to represent Male and Female. * Assay.Date - a column with strings to represent the batches in which the data was collected. * You will need a column for each trait of interest you wish to study.

Prior to the loading of the data. The trait data has been recoded to 0 to represent "as expected phenotypes"" and 1 to represent "not as expected phenotypes"". For example, if the options for a variable are normal or abnormal then normal would be replaced with 0 and abnormal with 1.

#Set the working directory of where to locate the data file
setwd("XXXXXXXXXXXXXXXXXXXXXX")
#Load the data file
data=read.csv("S1 Data.csv")
#List the traits of interest to study within this datafile
VariableList=c( "Bulging.Eye" , "Eye.Hemorrhage","Eyelid.Morphology" ,"Eyelid.Closure" ,"Narrow.Eye.Opening" , "Cornea", "Corneal.Opacity", "Corneal.Vascularisation", "Fusion.Between.Cornea.And.Lens", "Iris.Pupil" , "Synechia" , "Pupil.Position" , "Pupil.Shape","Pupil.Dilation" ,"Pupil.Light.Response" ,"Iris.Pigmentation", "Lens","Lens.Opacity" , "Retina" ,"Retinal.Pigmentation" , "Retinal.Structure" , "Optic.Disc" , "Retinal.Blood.Vessels" , "Retinal.Blood.Vessels.Structur", "Retinal.Blood.Vessels.Pattern" , "Persistence.Of.Hyaloid.Vascula")

## Functions for stage 1 testing

#Function to return MH_mid p value
#' Notations
#'
#' Females: Males:
#'
#' | |KO |WT | | | |KO |WT | |
#' |--|:--:|:--:|:--:| |--|:--:|:--:|:--:|
#' |1 |y_f | |yx_f| |1 |y_m | |yx_m|
#' |0 | | | | |0 | | | |
#' | |n2_f|n1_f| | | |n2_m|n1_m| |

#' Cochran-Mantel-Haenszel Test
#'
#' Tests the proprtion of rare events difference between the KO and WT groups, stratified by Sex.
#' The test is one-sided. Tests whether the KO group has larger proportion than the WT.
#'
#' @param y_f, yx_f, n2_f, n1_f, y_m, yx_m, n2_m, n1_m
#' Margins and values of the 2x2x2 contingancy table (see notations above)
#'
#' @param example
#' Logical. If TRUE the example from [*] is analysed (useful for debugging or demonstration).
#'
#' @return Numeric vector with 3 values :alpha-star, p-value and mid p-value.
#'
#' @example
#' MH_test(example=T)
#'
#' [*] book: Nonparametric Statistical Methods, 3e Hollander, Wolfe & Chicken, chapter 10)

MH_test <- function (y_f, yx_f, n2_f, n1_f, y_m, yx_m, n2_m, n1_m, example = F, alpha_star = "old")
{
 if (example)
 {
 z <- array(c(2, 1, 2, 5, 1, 5, 4, 1), dim=c(2, 2, 2))
 y_f <- z[1,1,1]
 n2_f <- sum(z[,1,1])
 n1_f <- sum(z[,2,1])
 yx_f <- sum(z[1,,1])
 y_m <- z[1,1,2]
 n2_m <- sum(z[,1,2])
 n1_m <- sum(z[,2,2])
 yx_m <- sum(z[1,,2])
 }
 else
 z <- array(c(y_f,n2_f-y_f,yx_f-y_f,n1_f-(yx_f-y_f),y_m,n2_m-y_m,yx_m-y_m,n1_m-(yx_m-y_m)), dim=c(2, 2, 2))

 # alpha star:
 # high limit of the support
 hi_vec <- c(min(n2_f,yx_f),min(n2_m,yx_m))

 if (alpha_star == "new")
 {
 z_as <- array(c(n2_f ,0 ,max(0,yx_f-n2_f) ,n1_f-max(0,yx_f-n2_f) ,
 n2_m ,0 ,max(0,yx_m-n2_m) ,n1_m-max(0,yx_m-n2_m) ), dim=c(2, 2, 2))
 }
 else
 {
 z_as <- array(c(hi_vec[1],n2_f-hi_vec[1],yx_f-hi_vec[1],n1_f-(yx_f-hi_vec[1]),
 hi_vec[2],n2_m-hi_vec[2],yx_m-hi_vec[2],n1_m-(yx_m-hi_vec[2])), dim=c(2, 2, 2))
 }
 as <- mantelhaen.test(x = z_as,alternative = "greater", exact = T)$p

 # p-value:
 z <- array(c(y_f,n2_f-y_f,yx_f-y_f,n1_f-(yx_f-y_f),y_m,n2_m-y_m,yx_m-y_m,n1_m-(yx_m-y_m)), dim=c(2, 2, 2))
 out <- mantelhaen.test(x = z,alternative = "greater", exact = T)
 pv <- out$p

 # mid-p-value:
 s <- out$s
 if (s==sum(hi_vec))
 mid_pv <- pv/2
 else
 {
 z_1 <- z
 if (z[1,1,1]<hi_vec[1])
 {
 z_1[1,1,1] <- z[1,1,1] + 1
 z_1[2,1,1] <- z[2,1,1] - 1
 z_1[1,2,1] <- z[1,2,1] - 1
 z_1[2,2,1] <- z[2,2,1] + 1
 }
 else
 {
 z_1[1,1,2] <- z[1,1,2] + 1
 z_1[2,1,2] <- z[2,1,2] - 1
 z_1[1,2,2] <- z[1,2,2] - 1
 z_1[2,2,2] <- z[2,2,2] + 1
 }
 pv_1 <- mantelhaen.test(x = z_1,alternative = "greater", exact = T)$p
 mid_pv <- (pv+pv_1)/2
 }
 out <- c(as,pv,mid_pv)
 names(out) <- c("alpha_star","pv","mid_pv")
 return(out)
}

## Functions for stage 2 testing

#Function to return the LR_KO alpha star value

#LR_KOalpha star p value which is the most extreme value possible arising as a function of the number of abnormal calls and number of readings taken.
##Using the annotation used within this code:
#' Females: Males:
#'
#' | |KO |WT | | | |KO |WT | |
#' |--|:--:|:--:|:--:| |--|:--:|:--:|:--:|
#' |1 |y_f | |yx_f| |1 |y_m | |yx_m|
#' |0 | | | | |0 | | | |
#' | |n2_f|n1_f| | | |n2_m|n1_m| |

##The 2 by 2 table for stage 2 would be:
#' | |KO _M |KO_F |
#' |--|:- -:|:--: |
#' |1 |y_m | y_f |
#' |0 | | |
#' | |n2_m |n2_f |

#Four arguments - all numeric values
#Example use LR_KOalphaStar(7,2,123,134)

LR_KOalphaStar<-function(y_m, y_f, n2_m, n2_f){

 #build a dataframe based on the most extreme values being loaded into the males
 if((y_m+y_f)<=n2_m){ #number of abnormalities is less than the number of male mice

 #' | |KO _M |KO_F |
 #' |--| | |
 #' |1 |(y_m +y_f ) | |
 #' |0 |(n2_m)-(y_m +y_f ) |n2_f |

 male_df <- data.frame(AbnormalityCall=c(rep(x=0, times=((n2_m)-(y_m +y_f ))), rep(x=1, times=(y_m +y_f ))), Sex=rep(x="Male", times=n2_m))
 female_df <- data.frame(AbnormalityCall=rep(x=0, times=n2_f), Sex=rep(x="Female", times=n2_f))
 male_extreme=rbind(male_df, female_df)

 }else { #number of abnormalities is greater than the number of male mice
 #if ((y_m+y_f)>n2_m)
 #' | |KO _M |KO_F |
 #' |--| | |
 #' |1 |n2_m |(y_m+y_f)-n2_m |
 #' |0 | |n2_f- ((y_m+y_f)-n2_m ) |

 male_df <- data.frame(AbnormalityCall=rep(x=1, times=n2_m), Sex=rep(x="Male", times=n2_m))
 female_df <- data.frame(AbnormalityCall=c(rep(x=1, times=((y_m+y_f)-n2_m)), rep(x=0, times=(n2_f- ((y_m+y_f)-n2_m )))), Sex=rep(x="Female", times=n2_f))
 male_extreme=rbind(male_df, female_df)
 }
 require(logistf)

 Signal2=length(levels(as.factor(male_extreme[ ,"AbnormalityCall"]))) ##assessing whether sufficient signal within the male_extreme table to allow model fitting

 if(Signal2==2){

 formula_full_stage2=model_Formula(modelType="full", depVariableString="AbnormalityCall") #returns the model formula Y~Sex
 model_full_stage2 <- do.call("logistf", args=list(formula_full_stage2, data=male_extreme, na.action="na.omit")) #fits the test model to the male extreme data
 Male_extreme_pval=logistftest(model_full_stage2)$prob # strategy needed to test effect as you cannot specify an intercept only model with logistf you cannot anova(test, null)

 }else{
 Male_extreme_pval=1 #when there is no abnormality signal returns a p value of 1
 }
 #build a dataframe based on the most extreme values being loaded into the females
 if((y_m+y_f)<=n2_f){ #number of abnormalities is less than the number of female mice

 #' | |KO _M |KO_F |
 #' |--| | |
 #' |1 | |(y_m +y_f ) |
 #' |0 |(n2_m) |n2_f-(y_m +y_f ) |
 female_df <- data.frame(AbnormalityCall =c(rep(x=0, times=(n2_f-(y_m +y_f ))), rep(x=1, times=(y_m +y_f ))), Sex=rep(x="Female", times=n2_f))
 male_df <- data.frame(AbnormalityCall =rep(x=0, times=n2_m), Sex=rep(x="Male", times=n2_m))
 female_extreme=rbind(male_df, female_df)

 }else { #number of abnormalities is greater than the number of female mice
 #if ((y_m+y_f)>n2_f)

 #' | |KO _M |KO_F |
 #' |--| | |
 #' |1 |(y_m+y_f)-n2_f |n2_f |
 #' |0 | n2_m-((y_m+y_f)-n2_f) | |

 female_df <- data.frame(AbnormalityCall=rep(x=1, times=n2_f), Sex=rep(x="Female", times=n2_m))
 male_df <- data.frame(AbnormalityCall=c(rep(x=1, times=((y_m+y_f)-n2_m)), rep(x=0, times=(n2_m-((y_m+y_f)-n2_f )))), Sex=rep(x="Male", times=n2_f))
 female_extreme=rbind(male_df, female_df)
 }

 Signal2=length(levels(as.factor(female_extreme[ ,"AbnormalityCall"]))) ##assessing whether sufficient signal within the female_extreme table to allow model fitting
 if(Signal2==2){
 formula_full_stage2=model_Formula(modelType="full", depVariableString="AbnormalityCall") #returns the model formula Y~Sex
 model_full_stage2 <- do.call("logistf", args=list(formula_full_stage2, data=female_extreme, na.action="na.omit")) #fits the test model to the female extreme data
 Female_extreme_pval=logistftest(model_full_stage2)$prob # strategy needed to test effect as you cannot specify an intercept only model with logistf you cannot anova(test, null)
 }else{
 Female_extreme_pval=1 #when there is no abnormality signal returns a p value of 1
 }
 output=min(Male_extreme_pval, Female_extreme_pval)
 #names(output) = "LR_KO_alphaStar"2
 return(output)
}

#Function to return formula for model fitting for the LR_KO stage 2 testing
#Two arguments
#First: modelType can be "full" or "null"
#Second: depVariableString is a string to represent the dependent Variable eg "Sodium"
#Example use model_Formula("full", "Sodium")

model_Formula <- function(modelType, depVariableString){

 if(modelType=="full"){
 model.formula <- as.formula(paste(depVariableString, "Sex", sep="~"))
 }else if(modelType=="null"){
 model.formula <- as.formula(paste(depVariableString, "~", "1", sep= " "))
 }
 return(model.formula)
}

#Function to return the stage two testing (pvalue, estimated coefficient and SE on estimated coefficient).
#Here we test the differences in abnormality rate between the male and female knockout data for a dependent variable using logistf
#Two arguments
#First is a dataframe with specification described above except the dataframe has been amended and only knockout data is presented
#Assumes the dataframe has a column called Sex and a column for the dependentVarible labelled with a string which is passed through as argument "depVariableString"

LR_KOdata<-function(KOdata, depVariableString){

 require(logistf)

 Signal2=length(levels(as.factor(KOdata[ ,depVariableString]))) ##assessing whether signal within the knockout mice to allow model fitting

 if(Signal2==2){

 formula_full_stage2=model_Formula(modelType="full", depVariableString) #returns the test model
 model_full_stage2 <- do.call("logistf", args=list(formula_full_stage2, data=KOdata, na.action="na.omit")) #fits the test model to the knockoutdata
 LR_KO_pval=logistftest(model_full_stage2)$prob # strategy needed to test effect as you cannot specify an intercept only model with logistf you cannot anova(test, null)
 finalmodel=summary(model_full_stage2)
 output=c(LR_KO_pval, finalmodel$coefficients[2], format(sqrt(diag(vcov(finalmodel))),scientific=FALSE)[2])
 names(output)=c("S2_LR_KO_pval", "S2_LR_KO_est_coeff", "S2_LR_KO_coeff_SE")
 }else{
 output=c(1, 0,0) #when there is no abnormality signal returns a p value of 1 and 0 for estimate and SE
 names(output)=c("S2_LR_KO_pval", "S2_LR_KO_est_coeff", "S2_LR_KO_coeff_SE")
 }
 return(output)
}

## Other functions used within the analysis

#This function ensures that the refGenotype (e.g."+/+") is set as the reference genotype for the analysis
#Two arguments:
#First: A dataframe with a Genotype column with two Genotypes
#Second: A string which indicates the refGenotype

EnsureWTisRefLevel<-function(dataset, refGenotype){
 Genotype_levels=levels(dataset$Genotype)

 if(sum(grepl(refGenotype, Genotype_levels, fixed=TRUE))==1){
 dataset$Genotype=relevel(dataset$Genotype, ref=refGenotype)
 return(dataset)
 }
}

#This function ensures that the there is sufficient data in each Genotype, Sex group
#Two arguments:
#First: A dataframe with a Genotype column with two Genotypes, a Sex column with two options (eg Male or Female)
#Second: A string which indicates the trait of interest

sufficentDataPerVariable<-function(dataset, depVariableString){
 require(plyr)
 countdata = ddply(.data=dataset, .variables=c("Genotype", "Sex"), .fun=countDataPoints, depVariableString)

 if(countdata[1,3]>2 && countdata[2,3]>2 && countdata[3,3]>2 && countdata[4,3]>2){
 value=TRUE
 }else{
 value=FALSE
 }
 return(value)
}

#This function counts the number of data points within a dataset for a column
#Two arguments:
#First: A dataframe
#Second: A string which indicates the column to look at.

countDataPoints<-function(dataset, depVariable){
 return(sum(is.finite(dataset[ , depVariable])))
}

## Statistical output for one dataset for one variable of interest

A wrapper function is use to wrap the above functions to calculate the various statistical output needed and to calculate a measure of biological effect for each stage for one dataset for one variable of interest

This function has 5 arguments:

- The first argument is for the data frame as described above
- The second argument is a string to indicate the testGenotype e.g. "Myo10/Myo10"
- The third argument is a string to indicate the refGenotype e.g. "+/+"
- the fourth argument is a string to indicate the batch column name e.g. "Assay.Date"
- The final argument is a string to indicate the column name of the trait of interest e.g. "Retinal.Structure"

Example usage: ImpactGenotypeSex_forSingleDataset(df=data,testGenotype= "Myo10/Myo10", refGenotype="+/+", BatchColumn ="Assay.Date", depVariableString="Retinal.Structure")

Output is a vector of length 23. Each point in the vector has been labelled to clearly identify content. S1 indicates output associated with stage 1 and S2 output associated with stage 2 testing.

ImpactGenotypeSex_forSingleDataset<-function(df,testGenotype, refGenotype, BatchColumn, depVariableString){
 require(vcd)
 require(grid)
 require(PhenStat)
 require(Epi)

 output=c()

 #Ensure refGenotype is reference level
 WTandKOdataset=EnsureWTisRefLevel(df, refGenotype)

 #use the phenStat framework (also allows the use of comparison to current method)
 #using the ZygosityColumn as the Genotype column as it will have WT or HET/HOM in the assembled data
 test=PhenList(df, testGenotype=testGenotype, refGenotype=refGenotype, dataset.clean=TRUE, dataset.colname.batch=BatchColumn, dataset.colname.genotype="Genotype", outputMessages=FALSE)

 # runs the PhenStat FE method to obtain a count matrix
 result=testDataset(phenList=test, depVariable=depVariableString, outputMessages=FALSE, pThreshold=0.05, method="FE", transformValues=FALSE)
 allCountMatrices <- getCountMatrices(result)
 #They are organised as matrix as below:
 #' Females: Males:
 #'
 #' | |WT |KO | | |WT |KO |
 #' |--|:-- |:--: |:--:| |:--:|:--:|
 #' |0 |[1,1] |[1,2] | |0 | | |
 #' |1 |[2,1] |[2,2] | |1 | | |
 #stage 1 testing: MH_test
 Stage1_MH=MH_test(y_f=allCountMatrices$female[2,2], yx_f=(allCountMatrices$female[2,2]+allCountMatrices$female[2,1]), n2_f=(allCountMatrices$female[2,2]+allCountMatrices$female[1,2]), n1_f=(allCountMatrices$female[1,1]+allCountMatrices$female[2,1]), y_m =allCountMatrices$male[2,2], yx_m=(allCountMatrices$male[2,2]+allCountMatrices$male[2,1]), n2_m=(allCountMatrices$male[2,2]+allCountMatrices$male[1,2]), n1_m =(allCountMatrices$male[1,1]+allCountMatrices$male[2,1]), example = F)
 names(Stage1_MH)=c("s1_MH_alpha_star", "S1_MH_pv", "S1_MH_midpv")

 #stage 1 testing:ES measure
 #Newcombe 95% CI for difference of two independent proportions
 #http://www.inside-r.org/packages/cran/epi/docs/ci.pd
 #Here the ES and CI is claculated for each table (male KO-WT and female KO-WT) separately. To give a summary ES for stage 1, which is a calculating the main effect across both tables. The average ES is calcualated and a CI selected by taking the min of the lower CI and the max of the upper CI from the two separate calculations. This would be a conservative estimate.

 CIoutput_Male=ci.pd(aa=allCountMatrices$male[2,1], bb=allCountMatrices$male[2,2], cc=allCountMatrices$male[1,1] ,dd=allCountMatrices$male[1,2], method = "Nc", alpha = 0.05, conf.level=0.95, digits = 3, print = FALSE, detail.labs = FALSE )
 CIoutput_Female=ci.pd(aa=allCountMatrices$female[2,1], bb=allCountMatrices$female[2,2], cc=allCountMatrices$female[1,1] ,dd=allCountMatrices$female[1,2], method = "Nc", alpha = 0.05, conf.level=0.95, digits = 3, print = FALSE, detail.labs = FALSE )
 CIoutput=c(CIoutput_Male[c(2, 4,5,6,7)],CIoutput_Female[c(2, 4,5,6,7)], (CIoutput_Male[5]+CIoutput_Female[5])/2, min(CIoutput_Male[6],CIoutput_Female[6]), max(CIoutput_Male[7],CIoutput_Female[7]) )
 names(CIoutput)=c("Male_AbnormalRateWT", "Male_AbnormalRateKO", "S1_DifferenceAbnormalRate_Male", "S1_lower95CI_Male", "S1_upper95CI_Male","Female_AbnormalRateWT", "Female_AbnormalRateKO", "S1_DifferenceAbnormalRate_Female", "S1_lower95CI_Female", "S1_Upper95CI_Female" , "S1_AvDiffAbnormalRate", "S1_lowerCI_acrossSexes", "S1_upperCI_acrossSexes")

 #stage 2 testing: Logistf regression comparing abnormality rates acrosses the sexes in knockout data only
 KOdatasetOnly=subset(result@analysedDataset, result@analysedDataset$Genotype!="WT") #prepare knockout data for stage 2 function
 Stage2_InteractionTest=LR_KOdata(KOdatasetOnly, depVariableString=depVariableString)

 LR_KOalphaStar=LR_KOalphaStar(y_m=allCountMatrices$male[2,2], y_f=allCountMatrices$female[2,2], n2_m=(allCountMatrices$male[2,2]+allCountMatrices$male[1,2]), n2_f=(allCountMatrices$female[2,2]+allCountMatrices$female[1,2]))
 names(LR_KOalphaStar) =c("S2_LR_KO_alphaStar")

 #CI for stage 2 test
 CI_stage2=ci.pd(aa=allCountMatrices$female[2,2], bb=allCountMatrices$male[2,2], cc=allCountMatrices$female[1,2] ,dd=allCountMatrices$male[1,2], method = "Nc",alpha = 0.05, conf.level=0.95, digits = 3, print = FALSE, detail.labs = FALSE )
 CI_stage2_output=c(CI_stage2[c(5,6,7)])
 names(CI_stage2_output)=c("S2_DiffAbnormalRate", "Stage2_lower95CI", "Stage2_upper95CI" )

 CombinedResults=c(CIoutput,Stage2_InteractionTest, Stage1_MH, CI_stage2_output, LR_KOalphaStar)

 return(CombinedResults)
}

results=ImpactGenotypeSex_forSingleDataset(df=data,testGenotype= "Myo10/Myo10", refGenotype="+/+", BatchColumn ="Assay.Date", depVariableString="Lens")

print(results)

## Male_AbnormalRateWT Male_AbnormalRateKO
## "0.0175131348511384" "0.428571428571429"
## S1_DifferenceAbnormalRate_Male S1_lower95CI_Male
## "-0.41105829372029" "-0.732087373025229"
## S1_upper95CI_Male Female_AbnormalRateWT
## "-0.140543945043757" "0.0440140845070423"
## Female_AbnormalRateKO S1_DifferenceAbnormalRate_Female
## "0.571428571428571" "-0.527414486921529"
## S1_lower95CI_Female S1_Upper95CI_Female
## "-0.797968731072092" "-0.206158659772004"
## S1_AvDiffAbnormalRate S1_lowerCI_acrossSexes
## "-0.46923639032091" "-0.797968731072092"
## S1_upperCI_acrossSexes S2_LR_KO_pval
## "-0.140543945043757" "0.000269774276234647"
## S2_LR_KO_est_coeff S2_LR_KO_coeff_SE
## "-0.874596885672597" "0.2505235"
## s1_MH_alpha_star S1_MH_pv
## "1.69788523186772e-22" "8.19016260624908e-08"
## S1_MH_midpv S2_DiffAbnormalRate
## "4.19754485204438e-08" "0.142857142857143"
## Stage2_lower95CI Stage2_upper95CI
## "-0.311063276859828" "0.525192004455133"
## S2_LR_KO_alphaStar
## "0.000126088132916813"

## Statistical output for one dataset for multiple variables of interest

A wrapper function to use the above functions and calculate a measure of biological effect for each stage for one dataset

This function has 5 arguments:

- The first argument is for the data frame as described above
- The second argument is a string to indicate the testGenotype e.g. "Myo10/Myo10"
- The third argument is a string to indicate the refGenotype e.g. "+/+"
- The fourth argument is a string to indicate the batch column name e.g. "Assay.Date"
- The final argument is a vector of strings to indicate the column name of the trait of interest e.g. variables where variables =c("Retinal.Structure", "Lens", "Cornea")

Example usage: ImpactGenotypeSex_forMultipleVariables_forDataset(df=data,testGenotype= "Myo10/Myo10", refGenotype="+/+", BatchColumn ="Assay.Date", depVariableString=VariableList)

Output: The function returns a dataframe

ImpactGenotypeSex_forMultipleVariables_forDataset<-function(df,testGenotype, refGenotype, BatchColumn, depVariableString, variablestToTest){

 output=c()

 for(bob in variablestToTest){

 if(sufficentDataPerVariable(df,bob)){

 tryCatch(
 {
 results=ImpactGenotypeSex_forSingleDataset(df=data,testGenotype= "Myo10/Myo10", refGenotype="+/+", BatchColumn ="Assay.Date", depVariableString=bob)
 metaData=c(testGenotype, refGenotype, bob)
 names(metaData)=c("Test_genotype", "Reference_genotype", "Variable_tested")

 #bind to earlier results
 output=rbind(results, output)
 },
 error=function(e){

 print(paste("This variable of interest failed to be processed", bob, sep=""))
 })
 }
 }
 return(output)
}

results2=ImpactGenotypeSex_forMultipleVariables_forDataset(df=data,testGenotype= "Myo10/Myo10", refGenotype="+/+", BatchColumn ="Assay.Date", variablestToTest=VariableList)

write.csv(results2, "results2.csv")
results2=read.csv("results2.csv")

## Managing the multiple testing and detecting significant phenotypes

Most studies monitor multiple traits, introducing a multiple testing burden. To manage multipel testing, but maximise sensitivity. The proposed methodology uses a mixture of potential filters, a false discovery rate (FDR) multiple testing adjustment and only adjusting the p values for stage 2 that were significant at stage 1. Due to the conservative nature and multiple testing burden, a more lentient FDR was proposed for stage 2.

In summary the proposed methodology is:

- For stage 1 BH correct only those MH alpha star <0.05
- For stage 2 BH correct only those LR_KO alpha star <0.05 and selected at stage 1
- FDR threshold: 5% FDR at stage 1 and 20% stage 2

In the code below, the methodology is implemented for this case example and it is used to generate a dataframe with new columns as detailed:

- column name = stage1__BH - this is the FDR adjusted p value for the stage 1 testing
- column name = stage2__BH - this is the FDR adjusted p value for the stage 2 testing
- column name = Classification1 - this has the options of "Significant at stage 1 but not 2", "not significant", and "significant at both stage 1 and 2"
- column name = Classification2 - this has the options of "Genotype effect at stage 1 only", "Males greater", "Females greater", and "No genotype effect"

This dataframe can be saved for further analysis. Within this code, we summarise the number of calls at various stages to understand the dataset.

options(width = 140)

PossSigStage1=subset(results2, results2$s1_MH_alpha_star<0.05)
PossSigStage1$stage1__BH=p.adjust(PossSigStage1$S1_MH_midpv, method="BH") #adjusting only those potential at stage 1
print(c("No. datasets tested", "=", nrow(results2)))

## [1] "No. datasets tested" "=" "26"

print(c("No. datasets that have potential at S1", "=", nrow(PossSigStage1[PossSigStage1$s1_MH_alpha_star<=0.05,])))

## [1] "No. datasets that have potential at S1" "=" "23"

NotPossSigStage1=subset(results2, results2$s1_MH_alpha_star>=0.05)
NotPossSigStage1$stage1__BH=rep(NA, length(NotPossSigStage1$s1_MH_alpha_star))
NotPossSigStage1$stage2__BH=rep(NA, length(NotPossSigStage1$s1_MH_alpha_star))
NotSigStage1=subset(PossSigStage1, PossSigStage1$stage1__BH>0.05)
NotSigStage1$stage2__BH=rep(NA, length(NotSigStage1$s1_MH_alpha_star))
SigStage1=subset(PossSigStage1, PossSigStage1$stage1__BH<=0.05)
PossSigStage2=subset(SigStage1, SigStage1$S2_LR_KO_alphaStar<=0.05)
print(c("No. datasets significant at S1", "=",nrow(SigStage1)))

## [1] "No. datasets significant at S1" "=" "16"

print(c("No. datasets possible significant at S2", "=",nrow(PossSigStage2)))

## [1] "No. datasets possible significant at S2" "=" "7"

PossSigStage2$stage2__BH=p.adjust(PossSigStage2$S2_LR_KO_pval, method="BH")
print(c("No.datasets significant at S2", "=",nrow(PossSigStage2[PossSigStage2$stage2__BH<=0.20,])))

## [1] "No.datasets significant at S2" "=" "7"

NotPossSigStage2=subset(SigStage1, SigStage1$S2_LR_KO_alphaStar>0.05)
NotPossSigStage2$stage2__BH=rep(NA, length(NotPossSigStage2$s1_MH_alpha_star))
recompiledData=rbind(NotPossSigStage2,PossSigStage2,NotPossSigStage1, NotSigStage1)

#return classification based on significance at the two step testing process

#Classifying effect by looking at both stage 1 and stage 2
FinalOutcomeV2<-function(stage1_adjustedPval, stage2_adjustedPval, Stage2_threshold, Stage1_threshold){
 if(is.na(stage1_adjustedPval)){
 outcome="not significant"
 }else if(stage1_adjustedPval <= Stage1_threshold & is.na(stage2_adjustedPval)){
 outcome="Significant at stage 1 but not 2"
 }else if(stage1_adjustedPval <= Stage1_threshold & stage2_adjustedPval > Stage2_threshold){
 outcome="Significant at stage 1 but not 2"
 }else if(stage1_adjustedPval <= Stage1_threshold & stage2_adjustedPval <= Stage2_threshold ){
 outcome=as.character("significant at both stage 1 and 2")
 }else if(stage1_adjustedPval > Stage1_threshold){
 outcome="not significant"
 }
 return(outcome)
}

#Add classification to the dataframe based on stage1 and stage 2 p values
recompiledData$Classification1=mapply(FUN=FinalOutcomeV2, stage1_adjustedPval=recompiledData$stage1__BH,stage2_adjustedPval=recompiledData$stage2__BH, Stage2_threshold=0.20, Stage1_threshold=0.05)
table(recompiledData$Classification1)

##
## not significant significant at both stage 1 and 2 Significant at stage 1 but not 2
## 10 7 9

#Summary statement
FinalOutcomeV3<-function(Classification,Stage2DiffAbRate){
 if(Classification=="not significant"){
 outcome="No genotype effect"
 }else if(Classification=="Significant at stage 1 but not 2"){
 outcome="Genotype effect at stage 1 only"
 }else if(Stage2DiffAbRate>0){
 outcome="Females greater"
 }else if(Stage2DiffAbRate<0){
 outcome="Males greater"
 }
 return(outcome)
}

recompiledData$Classification2=mapply(FUN=FinalOutcomeV3,Classification=recompiledData$Classification1, Stage2DiffAbRate=recompiledData$S2_DiffAbnormalRate)
table(recompiledData$Classification2)

##
## Females greater Genotype effect at stage 1 only No genotype effect
## 7 9 10

## Finally we capture the packages installed and versions used during this demonstration

sessionInfo()

## R version 3.3.1 (2016-06-21)
## Platform: i386-w64-mingw32/i386 (32-bit)
## Running under: Windows 7 x64 (build 7601) Service Pack 1
##
## locale:
## [1] LC_COLLATE=English_United Kingdom.1252 LC_CTYPE=English_United Kingdom.1252 LC_MONETARY=English_United Kingdom.1252
## [4] LC_NUMERIC=C LC_TIME=English_United Kingdom.1252
##
## attached base packages:
## [1] grid stats graphics grDevices utils datasets methods base
##
## other attached packages:
## [1] plyr_1.8.4 logistf_1.21 mgcv_1.8-13 nlme_3.1-128 mice_2.25 Rcpp_0.12.6 Epi_2.0 PhenStat_2.6.0 vcd_1.4-1
##
## loaded via a namespace (and not attached):
## [1] digest_0.6.10 htmltools_0.3.5 minqa_1.2.4 splines_3.3.1 MatrixModels_0.4-1 stringr_1.0.0 knitr_1.13
## [8] survival_2.39-5 lme4_1.1-12 nnet_7.3-12 lattice_0.20-33 cmprsk_2.2-7 Matrix_1.2-6 MASS_7.3-45
## [15] lmtest_0.9-34 zoo_1.7-13 stringi_1.1.1 pbkrtest_0.4-6 magrittr_1.5 car_2.1-3 rmarkdown_1.0
## [22] evaluate_0.9 colorspace_1.2-6 yaml_2.1.13 tools_3.3.1 parallel_3.3.1 nortest_1.0-4 nloptr_1.0.4
## [29] quantreg_5.26 formatR_1.4 etm_0.6-2 rpart_4.1-10 SparseM_1.7
