# Supplementary material for: Improving the Identification of Phenotypic Abnormalities and Sexual Dimorphism in Mice When Studying Rare Event Categorical Characteristics
Source: Genetics. 2016 Dec 5;205(2):491–501. doi: 10.1534/genetics.116.195388 (PMC5289831; doi:10.1534/genetics.116.195388)
Supplement: Supplementary file 14 [file 491TableS1.docx]

Table S1. Assessing for batch variation. (.xls, 49 KB)

[www.genetics.org/lookup/suppl/doi:10.1534/genetics.116.195388/-/DC1/TableS1.xls](http://www.genetics.org/lookup/suppl/doi:10.1534/genetics.116.195388/-/DC1/TableS1.xls)
